# DNA methylation mediates the immunosuppressive tumour microenvironment in metastatic endometrial clear cell carcinoma

**DOI:** 10.1016/j.ebiom.2025.105954

**Published:** 2025-09-30

**Authors:** Huiqing Jia, Yang Chen, Guofeng Ma, Sicong Xu, Xiangyan Zhang, Lianpeng Chang, Ping Yang, Yujing Xiao, Xuefeng Xia, Shukun Zhang, Huaxiao Tang, Yilin Mou, Lina Zhang, Haoyan Wang, Jing Bai, Xin Yi, Xiaoming Xing

**Affiliations:** a Department of Pathology, The Affiliated Hospital of Qingdao University, Qingdao, Shandong, China; b Department of Urology, The Affiliated Hospital of Qingdao University, Qingdao, Shandong, China; c GenePlus-Beijing Institution, Beijing, China; d GenePlus-Shenzhen Institution, Shenzhen, Guangdong, China; e Department of Pathology, Affiliated Yantai Yuhuangding Hospital of Qingdao University, Yantai, Shandong, China; f Department of Pathology, Weihai Municipal Hospital, Weihai, Shandong, China; g Department of Pathology, The Affiliated Weihai Second Municipal Hospital of Qingdao University, Weihai, Shandong, China; h Department of Pathology, Linyi People's Hospital, Linyi, Shandong, China; i Department of Pathology, Heze Dingtao District People's Hospital, Heze, Shandong, China; j College of Future Technology, Peking University, Beijing, China

**Keywords:** Endometrial clear cell carcinoma, DNA methylation, Immunosuppressive tumour microenvironment, ETS1 regulon, *LCK*, Metastatic mechanisms

## Abstract

**Background:**

Endometrial clear cell carcinoma (ECCC) is a rare and highly aggressive histological subtype of endometrial cancer with marked metastatic potential. The molecular characteristics and underlying mechanisms governing its metastatic behaviour remain poorly understood. This study aimed to delineate molecular distinctions between metastatic (Pm) and non-metastatic (Pn) primary ECCC tumours, elucidate DNA methylation-mediated regulatory mechanisms driving metastasis, and identify potential epigenetic biomarkers and therapeutic targets.

**Methods:**

This multicentre study involved 51 individuals diagnosed with ECCC, leading to the establishment of two independent cohorts: a sequencing cohort (n = 35) for integrated whole-genome methylation and transcriptomic analysis, and a tissue microarray (TMA) cohort (n = 16) to validate key findings.

**Findings:**

Tumours exhibiting metastasis were found to possess a profoundly immunosuppressive tumour microenvironment (TME), evidenced by reduced density of tumour-infiltrating lymphocytes (TILs), especially within subsets of anti-tumour immune cells. Further analysis highlighted differential hypermethylation events in Pm tumours that acted as crucial epigenetic switches regulating immune responses. Specifically, methylation at ETS1-binding sites influenced ETS1 regulon activity, thus broadly regulating immune response processes. Epigenetic silencing of key genes in the T cell receptor (TCR) signalling pathway, such as *LCK*, *CD3E*, and *ZAP70*, impaired T cell activation and inhibited the activity of interacting immune pathways. Additionally, we developed a Lasso-derived metastatic risk score model, incorporating TME features (TIL density) and epigenetic predictors (*LCK* methylation), which demonstrated strong predictive performance (area under the curve [AUC] = 0.859).

**Interpretation:**

This study illuminated the “epigenetic-immune axis” as a central regulatory mechanism driving ECCC metastasis. DNA methylation systematically silenced immune response genes by targeting ETS1-binding sites and TCR signalling components, thus reconstructing the immunosuppressive TME to facilitate metastasis. The development of the metastatic risk score model and identification of *LCK* as a potential therapeutic target provide valuable strategies for precision treatment decisions and advancing targeted epigenetic-immune therapies in ECCC.

**Funding:**

This work was supported by the 10.13039/501100001809National Natural Science Foundation of China, Joint Foundation Programme, Qingdao Municipal Science and Technology Bureau Municipal Science, Shenzhen Science and Technology Programme, and the Affiliated Hospital of Qingdao University Young Investigator Fund.

**Research in context**

**Evidence before this study**

Endometrial clear cell carcinoma (ECCC), a rare variant of endometrial cancer, poses significant clinical challenges due to its high metastatic potential and severely poor prognosis. However, the molecular mechanisms propelling ECCC metastasis remain inadequately understood, and specific biomarkers for risk stratification or therapeutic targeting in ECCC are absent. While DNA methylation's role in tumour progression is recognised, comprehensive analyses linking whole-genome methylation profiles to the metastatic phenotype in ECCC have not been conducted, and the precise regulatory mechanisms by which DNA methylation influences ECCC metastasis remain unclear.

**Added value of this study**

This research unveils unique molecular attributes of primary tumours in metastatic ECCC, enhancing understanding of the regulatory role of DNA methylation in ECCC metastasis. We pinpointed specific DNA methylation alterations crucial in shaping the immunosuppressive tumour microenvironment (TME), elucidating the principal “epigenetic-immune axis” mechanism underpinning ECCC metastasis. Briefly, DNA methylation remodels the immunosuppressive TME by targeting ETS1-binding sites and key genes in the T cell receptor (TCR) signalling pathway, spurring tumour metastasis. Building on these discoveries, we created a metastatic risk score model that integrates TME features (TIL density) and epigenetic predictors (*LCK* methylation), and identified *LCK* as a significant epigenetic regulatory target for predicting ECCC metastasis and potential therapy.

**Implications of all the available evidence**

This research provides an epigenetic perspective on the immune regulatory mechanisms involved in ECCC metastasis. The metastatic risk score model could facilitate early clinical risk stratification and guide personalised treatment decisions. The discovery of epigenetic regulatory targets, including ETS1-binding sites and TCR signalling pathway genes, illuminates pathways involved in reprogramming the immunosuppressive TME, facilitating the development of promising therapies targeting epigenetic modifications and immunotherapy. These findings offer indispensable theoretical backing for advancing precision medicine in ECCC.

## Introduction

Endometrial clear cell carcinoma (ECCC) is an uncommon histological subtype of endometrial cancer, accounting for 2%–5% of all cases.^1^ Traditionally classified as a “Bokhman” type II tumour, ECCC is characterised by high-grade histology, frequent deep myometrial invasion, and lymphovascular space invasion (LVSI).^2^^,^^3^ Compared to stage-matched endometrioid carcinoma, ECCC exhibits a greater proclivity for extrauterine dissemination and metastasis. Around 40%–50% of individuals with early-stage localised disease eventually progress to metastatic disease,^4^^,^^5^ resulting in a steep decline in the 5-year overall survival (OS) rate to less than 25% among those with metastases.^6^ This highlights the critical need for early risk stratification. Comprehensive surgical staging is the primary strategy for early-stage disease. Although adjuvant chemotherapy and radiotherapy are employed to enhance outcomes, the efficacy of conventional therapies in preventing metastasis remains limited. In recent years, the utilisation of molecular subtypes for prognostic stratification and guiding treatment decisions in endometrial cancer has gained widespread recognition. Studies indicate that over 85% of ECCC cases correspond to prognostically unfavourable molecular subtypes: the no specific molecular profile (NSMP) or p53 abnormal (p53abn) subtypes.^7^^,^^8^ Crucially, tumours within these subtypes often exhibit reduced responsiveness to immune checkpoint inhibitors (ICB) due to inherently low immunogenicity, hindering patients from receiving any substantial benefit.^9^ Currently, mechanisms driving ECCC metastasis remain poorly elucidated, presenting clinicians with the dual challenge of lacking effective predictive biomarkers and specific therapeutic strategies.

DNA methylation is a key epigenetic regulatory mechanism offering unique advantages in early cancer detection and targeted therapy due to its reversible nature, serving both as a predictive biomarker and as a target for epigenetic intervention.^10^ Previous research has affirmed that dysregulation of genomic methylation plays an essential role in the development of endometrial cancer.^11^^,^^12^ MLH1 promoter methylation is a pivotal mechanism causing mismatch repair deficiency (MMRd).^13^ Hypermethylation of promoter regions can silence tumour suppressor genes or immune modulators, thereby promoting tumour immune evasion, while global hypomethylation may activate oncogenes, supporting tumour proliferation and invasion.^14^ Yet, the genome-wide methylation signature and its regulatory mechanisms driving ECCC metastatic progression remain largely unexplored, and strategies to target epigenetic nodes to block metastasis and counteract immunotherapy resistance are urgently required.

This study integrated data from 51 individuals with ECCC from multiple centres to create two independent research cohorts. The sequencing cohort (n = 35) underwent genome-wide methylation sequencing (GM-seq) and RNA sequencing (RNA-seq). Multi-omics integration analysis was conducted to systematically characterise the molecular differences between primary tumours from individuals who developed metastasis (Pm) and those who did not (Pn). The tissue microarray (TMA) cohort (n = 16) was used to validate key findings. The study aimed to achieve three central objectives: (1) elucidate distinct molecular features between Pm and Pn tumours; (2) uncover the core regulatory mechanisms by which DNA methylation drives ECCC metastasis; and (3) identify clinically relevant epigenetic biomarkers and therapeutic targets with translational potential. This work is expected to bridge the current gap in understanding the metastatic mechanisms of ECCC, offering a theoretical foundation for early risk stratification, personalised treatment, and research-based targeted epigenetic-immune modulation therapies.

## Methods

### Patients and samples

This retrospective study collected formalin-fixed paraffin-embedded (FFPE) tumour samples from 51 individuals diagnosed with ECCC who underwent complete surgical resection from January 2017 to March 2024 at six participating centres: the Affiliated Hospital of Qingdao University, the Affiliated Yantai Yuhuangding Hospital of Qingdao University, Weihai Municipal Hospital, the Affiliated Weihai Second Municipal Hospital of Qingdao University, Linyi People's Hospital, and Heze Dingtao District People's Hospital. Detailed clinicopathological data were systematically recorded for each participant, with sex information self-reported. All participants self-identified as Han Chinese, a classification aligned with the ethnic categorisation framework used in China. Follow-up data were obtained through structured reviews of electronic health records supplemented by standardised telephone interviews. OS served as the primary endpoint, defined as the interval from initial surgery to death from any cause or the date of last confirmed survival (follow-up cutoff: 31 August 2024). All participants completed follow-up, with comprehensive baseline characteristics documented in Table 1.

**Table 1:** Demographic and clinical characteristics of individuals with endometrial clear cell carcinoma (ECCC).

Pn, non-metastatic primary tumour; Pm, metastatic primary tumour; GM-seq, genome-wide methylation sequencing; RNA-seq, RNA sequencing; TILs,tumour-infiltrating lymphocytes; TLS, tertiary lymphoid structures; MMR, mismatch repair; MMRd, mismatch repair-deficient; p53abn, p53 abnormal; NSMP, no specific molecular profile; ER, oestrogen receptor; PR, progesterone receptor; LVSI, lymphovascular space invasion; PLN, pelvic lymph nodes; PaLN, para-aortic abdominal lymph nodes; PLND, pelvic lymphadenectomy; PaLND, para-aortic lymphadenectomy; THBSO, total hysterectomy with bilateral salpingo-oophorectomy; VBT, vaginal brachytherapy; ERBT, external beam radiation therapy.

^a^Five cases were excluded due to failure to meet quality control standards

### Ethics

The study protocol received approval from the Ethics Committee of the Affiliated Hospital of Qingdao University (QYFY WZLL 29487), and the requirement for informed consent was waived according to the study's observational design.

### Pathological review

All ECCC samples underwent independent blinded re-evaluation by three pathologists. Diagnosis adhered to WHO criteria, relying on characteristic histomorphological features: polygonal or hobnail tumour cells with clear or eosinophilic cytoplasm, and papillary, tubulocystic, or solid growth patterns. Histological confirmation of pure ECCC required: (1) evaluation of haematoxylin and eosin (H&E)-stained sections, supplemented by (2) immunohistochemical (IHC) analysis for HNF1β and Napsin A when necessary. All slides were converted into whole slide images (WSI) using a digital slide scanner (Win series, DANJIER, China). Pathological quality control confirmed a tumour cell content of ≥80% in all samples.

### TMA construction

Representative tumour regions were precisely localised by correlating FFPE blocks of ECCC samples with their corresponding WSI. Tissue cores (3 mm diameter) were extracted using a punch needle and systematically arrayed into a blank recipient paraffin block according to predefined spatial matrices. The cores were subsequently embedded into a unified TMA block using a tissue microarrayer (Servicebio-JX-10, Servicebio, China).

### RNA extraction and sequencing

Total RNA was isolated from FFPE samples using the RNeasy FFPE Kit (QIAGEN, Cat. No. 73504, Germany) RNA sequencing (RNA-seq) libraries were prepared with the NEBNext® Ultra™ II Directional RNA Library Prep Kit for Illumina® (New England Biolabs, Cat. No. E7760L, USA) following the manufacturer's protocol. Libraries underwent paired-end sequencing (2 × 150 bp) on the DNBSEQ-T7 platform (MGI, China). Raw sequencing data underwent quality control with fastp (v0.20.0),^15^ and ribosomal RNA was removed using bowtie2 (v2.3.5.1).^16^ Processed reads were aligned to the human reference genome (GRCh37/hg19) using STAR (v2.7.6a),^17^ followed by transcriptome assembly with StringTie2 (v2.0.4).^18^

### DNA extraction and GM-seq

Genomic DNA (gDNA) was isolated from FFPE samples using the FireGene FFPE gDNA Extraction Kit (FireGene, China). Subsequent GM-seq library preparation and sequencing were performed by Geneplus (Beijing, China) following an established protocol.^19^ For library construction, the Hieff NGS® Ultima Pro DNA Library Prep Kit for Illumina® (Yeasen Biotechnology, Cat. No. 12197ES96, China) was employed for end repair, A-tailing, and adaptor ligation according to manufacturer's instructions.^19^ Purified products were then subjected to magnetic bead-based oxidation of 5-methylcytosine (5 mC) and 5-hydroxymethylcytosine (5hmC) to 5-formylcytosine (5 fC) or 5-carboxylcytosine (5caC) via ten-eleven translocation (TET) enzyme catalysis, followed by reduction to dihydrouracil (DHU) using pyridine borane. PCR amplification was performed using indexed primers to incorporate sample-specific barcodes and generate final sequencing libraries. Through this process, methylated cytosines were converted to thymine (T) residues, enabling detection during sequencing on the DNBSEQ-T7 platform (MGI, China). Raw sequencing data were quality-filtered using fastp (v0.20.0)^15^ to remove low-quality reads, in accordance with quality control thresholds of Q20 ≥ 95% and Q30 ≥ 85%. The resulting clean reads were subsequently aligned to the GRCh37/hg19 reference genome using Sentieon (v202010) to generate BAM files. The alignment output was evaluated with Samtools (v1.9)^20^ to ensure a mapping rate of ≥90% and a duplication rate of ≤20%. Cytosine-phosphate-guanine (CpG) site detection was performed via the call function in asTair (v3.3.2), retaining only sites with a sequencing depth of ≥15× for downstream analysis.

### POLE sanger sequencing

Sanger sequencing was performed on tumour tissues from 51 individuals with ECCC to detect hotspot mutations within the exonuclease domain (exons 9–14) of the POLE gene. Target-specific primers (sequences in Supplementary Table S1) were employed for amplification with 2× Taq PCR MasterMix (AGBio, China) on an Applied Biosystems™ ProFlex™ PCR System (Thermo Fisher Scientific, Cat. No. 4484057, USA). The thermal cycling protocol comprised: initial denaturation at 95 °C for 5 min; 30 cycles of denaturation at 95 °C for 30 s, annealing at 60 °C for 30 s, and extension at 72 °C for 45 s; followed by final extension at 72 °C for 7 min. Amplified products were verified by 2% agarose gel electrophoresis and purified using the QIAquick PCR Purification Kit (QIAGEN, Cat. No. 28106, Germany). Bidirectional sequencing was conducted with the Applied Biosystems BigDye™ Terminator v3.1 Cycle Sequencing Kit (Thermo Fisher Scientific, Cat. No. 4337455, USA) on an ABI 3730xl DNA Analyser (Thermo Fisher Scientific, USA). Raw sequencing data were aligned to the reference sequence (NM_006231.4) using Chromas (v2.6.6), analysing the following 11 hotspot mutations: p.P286R, p.V411L, p.S297F, p.A456P, p.S459F, p.F367S, p.L424I, p.M295R, p.P436R, p.M444K, and p.D368Y.^21^ Tumours harbouring any such mutations were defined as POLE-mutated (POLE-mut) tumours.

### Partial least squares discriminant analysis (PLS-DA)

To evaluate transcriptomic differences between Pm and Pn tumours, we used PLS-DA for supervised classification,^22^ implemented in the ropls R package (v1.34.0). Standardised transcriptomic data were analysed by extracting principal components (PC1, PC2) to construct a score plot, with a 95% confidence ellipse added to quantify group distribution overlap. Model stability was assessed via 1000 permutation tests (empirical p-value <0.05), and generalisation ability was evaluated using 10-fold cross-validation (Q^2^ = 0.47).

### Analysis of differentially expressed genes (DEGs)

Analysis of differential gene expression was performed on raw RNA-seq count data using the limma R package (v3.58.1, RRID:SCR_010943). Significantly DEGs were defined by thresholds of |log2 (fold change) | ≥ 1 and p-value <0.05 (derived from an empirical Bayes moderated t-test).

### Functional enrichment analysis

Functional enrichment analyses were conducted using Metascape (http://metascape.org/gp/index.html#/main/step1).^23^ The “Custom Analysis” module was applied with parameters: minimum overlap ≥3 genes; p-value <0.01; and minimum enrichment factor ≥1.5. p-values were derived from a hypergeometric test and adjusted for multiple comparisons using the Benjamini-Hochberg method. Gene set databases included: Gene Ontology (GO) Molecular Functions, GO Biological Processes, GO Cellular Components, Kyoto Encyclopedia of Genes and Genomes (KEGG) Pathway, Hallmark Gene Sets, Reactome Gene Sets, and WikiPathways (RRID:SCR_002134).

### Gene set variation analysis and single-sample gene set enrichment analysis

To evaluate pathway activities and quantify tumour metastasis-associated signatures alongside immune microenvironment signatures in individuals with ECCC, we performed gene set variation analysis (GSVA) and single-sample gene set enrichment analysis (ssGSEA) on gene expression data using the GSVA R package (v1.50.1).^24^ Immune scores were derived via the ESTIMATE algorithm.^25^ Predefined gene sets were sourced from: the MSigDB database (https://www.gsea-msigdb.org/gsea/msigdb/index.jsp), TISIDB (http://cis.hku.hk/TISIDB/),^26^ and published signatures by Bagaev et al.,^27^ An et al.,^28^ Miranda et al.,^29^ and Peng et al.^30^

### Differential methylation analysis

Differentially methylated cytosines (DMCs) and regions (DMRs) were identified from ECCC methylation data using metilene (v0.2-8).^31^ DMR calling parameters included: minimum per-CpG coverage depth of 5×; maximum distance between adjacent CpGs within a DMR of 300 bp, minimum of 10 CpG sites per DMR; mean absolute methylation difference (|δβ|) ≥ 0.1; and significance threshold of p < 0.05 (derived from Wilcoxon rank-sum test). Identified DMRs were annotated with the methylKit R package (v1.16.1, RRID:SCR_005177),^32^ retaining those mapped to protein-coding genes.

### Motif enrichment analysis

Motif enrichment analysis for hypermethylated differentially methylated regions (DMRs) was performed using the findMotifsGenome.pl function in HOMER (v4.11, RRID:SCR_010881)^33^ with default parameters (genomic window size: 200–600 bp). Identified motifs were annotated via the annotatePeaks.pl function based on position weight matrices (PWMs).

### Transcriptional regulatory network analysis

In transcriptional regulatory network analysis, a regulon is defined as a functionally coherent set of target genes that are specifically regulated by a single transcription factor (TF), with their expression patterns co-ordinated by this TF.^34^ In this study, we constructed the transcriptional regulatory network using standardised gene expression data from patients with ECCC, employing the RTN R package (v2.26.0). To quantify the activity of each regulon—reflecting the overall regulatory influence of its corresponding TF on target genes—we calculated regulon activity scores using the tni.gsea2 function in the RTN R package via two-tailed gene set enrichment analysis (GSEA). Higher scores indicate a more pronounced regulatory effect of the TF on its target genes. The resulting activity scores can be directly used for subsequent differential comparison analyses.

### Survival analysis

OS was estimated using the Kaplan–Meier method. Inter-group differences were assessed by log-rank tests, with the survival R package (v3.5) for modelling and survminer (v0.4.9) for curve visualisation.

### Assessment of tumour-infiltrating lymphocytes and tertiary lymphoid structures

Tumour-infiltrating lymphocytes (TIL) and tertiary lymphoid structures (TLS) were assessed using WSI. TIL evaluation followed the standardised methodology by the International Immuno-Oncology Biomarkers Working Group.^35^ TIL density was defined as the percentage of tumour-associated stromal area occupied by stromal lymphocytes, assessed across entire tissue sections. TLS, characterised as rounded lymphocyte aggregates with mature morphology (defined by distinct germinal centre structures), were identified in samples from individuals with ECCC.

### IHC staining

IHC staining was performed on 4-μm-thick FFPE tissue sections. Primary antibodies included: p53 (Cat#60283-2-Ig, RRID:AB_2881401, Proteintech, China), MLH1 (Cat#11697-1-AP, RRID:AB_2145604, Proteintech, China), PMS2 (Cat#66075-1-Ig, RRID:AB_11182595, Proteintech, China), MSH2 (Cat#60161-1-Ig, RRID:AB_10666855, Proteintech, China), and MSH6 (Cat#66172-1-Ig, RRID:AB_2881567, Proteintech, China). For these markers, automated staining was conducted on the Aliya-B4 system (Talent Biomedical, China) per manufacturer protocols. For manual ETS1 staining: sections were dewaxed, rehydrated, and underwent heat-induced antigen retrieval in Tris–EDTA buffer (pH 9.0). Endogenous peroxidase activity was blocked with 3% hydrogen peroxide for 10 min, followed by incubation with primary ETS1 antibody (Cat#66598-1-Ig, RRID: AB_2881958, Proteintech, China) overnight at 4 °C. Horseradish peroxidase-conjugated secondary antibodies were applied, with 3,3′-diaminobenzidine (DAB) as chromogen. Nuclei were counterstained with haematoxylin.

### Immunohistochemical interpretation and analysis

p53 IHC interpretation strictly adhered to guidelines from the International Society of Gynaecological Pathologists (ISGyP).^36^ Tumour cell p53 staining patterns were categorised as: normal (wild-type), complete absence, overexpression, or cytoplasmic expression—with the latter three indicating abnormal (mutated) status. For mismatch repair (MMR) protein IHC, expression of four proteins (MLH1, MSH2, MSH6, PMS2) in tumour cells was evaluated.^37^ Normal nuclear staining denoted MMR proficiency (MMRp), while loss of any protein indicated MMR deficiency (MMRd). ETS1 expression in immune and tumour cells was quantified using the H-score method: H-score = Σ (Pi × i), where Pi represents the percentage of positive cells at intensity grade i (0: absent; 1: weak; 2: moderate; 3: strong), resulting in a range of 0–300.

### Molecular subtypes

Molecular subtypes of ECCC were determined in accordance with the WHO Classification of Female Genital Tumours (5th edition) (https://tumourclassification.iarc.who.int). This integrated analysis assessed POLE gene mutation status, MMR protein expression, and p53 expression to categorise tumours into four subtypes: POLE-mut, MMRd, p53abn, and NSMP. The subtyping algorithm adhered to a hierarchical approach: tumours with pathogenic POLE mutations were first classified as POLE-mut; for cases without POLE mutations, MMR protein expression was evaluated, with loss of any protein defining the MMRd subtype; among individuals without POLE mutations and with proficient MMR, abnormal p53 expression designated the p53abn subtype; whereas normal p53 expression indicated NSMP.

### Multiple immunofluorescence staining and analysis

Multiple immunofluorescence (mIF) staining was performed on FFPE sections. Sections were dewaxed, rehydrated, and subjected to heat-induced antigen retrieval. Following this, sections were treated with a 3% hydrogen peroxide solution for 10 min to block endogenous peroxidase activity. Sequential incubation with primary antibodies was then conducted according to the Opal staining protocol (Akoya Biosciences, USA). Antibodies were organised into two panels: Panel 1 comprised pan-cytokeratin (pan-CK) (Cat#26411-1-AP, RRID: AB_2880505, Proteintech, China) and CD45 (Cat#20103-1-AP, RRID: AB_2716813, Proteintech, China); Panel 2 comprised CD3 (Cat#Ab16669, RRID: AB_443425, Abcam, UK), CD4 (Cat#67786-1-Ig, RRID: AB_2918550, Proteintech, China), CD8 (Cat#Ab199016, RRID: AB_2860566, Abcam, UK), CD20 (Cat#Ab64088, RRID: AB_1139386, Abcam, UK), and CD56 (Cat#99746S, RRID:AB_2918374, Cell Signalling Technology, USA). Following incubation with horseradish peroxidase-labelled secondary antibodies, nuclei were counterstained with DAPI (Akoya Biosciences, USA). Multichannel imaging was performed using a Vectra Polaris imaging system (Akoya Biosciences, USA), and subsequent image analysis and quantification were conducted using QuPath software (v0.5.1, Queen's University Belfast, UK).^38^ Positive cells were quantified within a 2 mm^2^ area at 40× magnification across five randomly selected cellular enrichment regions (hotspots), with the mean value serving as the representative metric for each cellular subpopulation.

### Construction of the metastasis risk score model

To enhance model robustness, a staged variable selection strategy was employed during model development. Initially, metastasis-associated predictors were identified through univariate logistic regression analysis using the stats R package (v4.3.3), with Wald tests applied to assess variable significance (p-value). The top four predictors were selected based on p-value ranking. This step adhered to the conventional 1:10 variable-to-sample ratio principle (i.e., the number of variables was constrained to be as close as possible to one-tenth of the sample size). Subsequently, variable selection and regularisation were carried out using the least absolute shrinkage and selection operator (LASSO) regression with the glmnet R package (v4.1-8). The optimal penalty coefficient (lambda.1se), determined through five-fold cross-validation, selected the most parsimonious model within one standard error to maximise generalisability. The final model included two predictors: TIL density and LCK methylation β-value, formulated as follows: Risk score = −1.5487 + (−0.0028 × TIL density) + (2.3239 × LCK β-value). The model's discriminatory performance was evaluated using receiver operating characteristic (ROC) curves generated by the pROC R package (v1.18.5), with the area under the curve (AUC) quantification.

### Statistics

A minimum sample size of 50 individuals with ECCC was targeted to ensure adequate representation of molecular and clinical characteristics in this rare tumour cohort. This decision was informed by prior studies on similar rare cancers, balancing feasibility with statistical power. Given the retrospective nature of the study and reliance on existing clinical data, formal randomisation and blinding were not feasible. Inclusion criteria required histopathological confirmation of primary ECCC, while exclusion criteria encompassed patients with a history of hereditary gynaecological malignancies, prior neoadjuvant therapy, or concurrent primary ovarian carcinoma. Statistical analyses adhered to the following principles: categorical variables were compared using χ^2^ tests or Fisher's exact tests (for expected cell counts <5), while continuous variables were analysed with non-parametric tests (Mann–Whitney U for pairwise comparisons, Kruskal–Wallis H for multiple groups) due to the small sample size and non-normal distribution. Correlations between variables were assessed using Spearman's rank correlation coefficients. All hypothesis tests were two-tailed, with statistical significance defined as p ≤ 0.05. Analyses were conducted using R software (v4.3.4) and GraphPad Prism (v9.5.1, RRID:SCR_002798). Post-hoc power calculations confirmed ≥80% statistical power for all tests, mitigating concerns about underpowered inferential statistics.

### Role of funders

The funder of the study provided financial support but was not involved in the study design, data collection, analysis, interpretation, or manuscript preparation.

## Results

### Study cohort characteristics

**Fig. 1:**
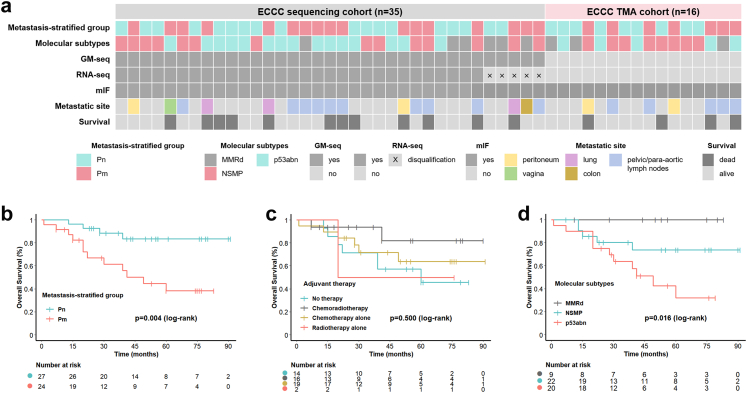
**Cohort characterisation and survival analysis in individuals with ECCC**. a. Overview of the study cohort design, depicting two independent cohorts: the ECCC sequencing cohort (n = 35) and the ECCC TMA cohort (n = 16). The key below denotes cohort characteristics including metastasis-stratified group, molecular subtype, multi-omics data availability, metastatic sites, and survival status. b–d. Kaplan–Meier OS analyses of the overall ECCC cohort (n = 51). b. Stratification by metastasis group (Pn, n = 27; Pm, n = 24; p = 0.004). c. Stratification by type of adjuvant therapy received (No therapy, n = 14; Chemoradiotherapy, n = 16; Chemotherapy alone, n = 19; Radiotherapy alone, n = 2; p = 0.500). d. Stratification by molecular subtype (MMRd, n = 9; NSMP, n = 22; p53abn, n = 20; p = 0.016). All p-values were determined by log-rank test. ECCC, endometrial clear cell carcinoma; TMA, tissue microarray; GM-seq, genome-wide methylation sequencing; RNA-seq, RNA sequencing; mIF, multiplex immunofluorescence; Pn, non-metastatic primary tumours; Pm, metastatic primary tumours; MMRd, mismatch repair deficient; p53abn, p53 abnormal; NSMP, no specific molecular profile; OS, overall survival.

**Supplementary Figure S1:**
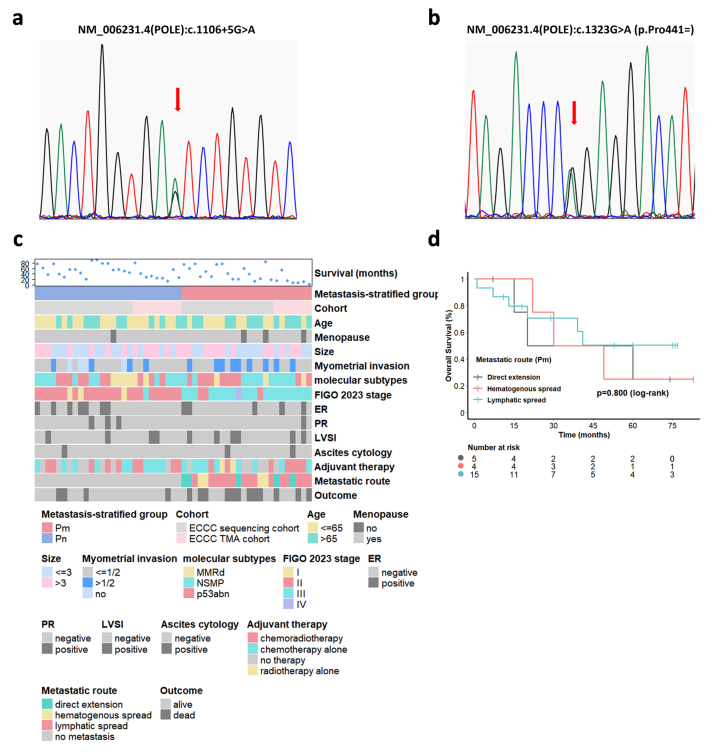
Cohort characterisation and survival analysis in individuals with ECCC, related to Fig. 1. a. Sanger sequencing chromatogram of the POLE variant c.1106+5G > A; the red arrow indicates the mutant base. b. Sanger sequencing chromatogram of the POLE variant c.1323G > A (p.Pro441=); the red arrow indicates the mutant base. c. Heatmap of clinicopathological characteristics stratified by metastasis status. The key below denotes clinicopathological features. d. Kaplan–Meier analysis of OS by route of metastasis (Direct extension, n = 5; Haematogenous spread, n = 4; Lymphatic spread, n = 15; p = 0.800, log-rank test). ECCC, endometrial clear cell carcinoma; TMA, tissue microarray; Pn, non-metastatic primary tumours; Pm, metastatic primary tumours; MMRd, mismatch repair deficient; p53abn, p53 abnormal; NSMP, no specific molecular profile; ER, oestrogen receptor; PR, progesterone receptor; LVSI, lymphovascular space invasion; OS, overall survival.

This multicentre study enrolled 51 individuals diagnosed with primary ECCC and established two independent cohorts: the sequencing cohort (n = 35, 68.6%) and the TMA cohort (n = 16, 31.4%). The cohort design is summarised in Fig. 1a. The median age of the overall cohort was 65 years (range: 38–76), with a median tumour diameter of 3 cm (range: 0.7–12 cm). Among these individuals, 92.2% (47/51) were postmenopausal. Regarding molecular subtypes, Sanger sequencing of the POLE gene identified two variants: c.1106+5G > A (Fig. S1a) and c.1323G > A (Fig. S1b). Both variants were classified as non-pathogenic; consequently, no tumours were categorised as POLE-mut subtype. The predominant subtypes were NSMP (43.1%, 22/51) and p53abn (39.2%, 20/51), followed by MMRd (17.7%, 9/51). All individuals underwent total hysterectomy with bilateral salpingo-oophorectomy; 96.1% (49/51) additionally received pelvic and/or para-aortic lymphadenectomy. Adjuvant therapy was received by 72.5% of individuals with ECCC (37/51), with chemotherapy alone being the most common regimen (51.4%, 19/37), followed by chemoradiotherapy (43.2%, 16/37), and radiotherapy alone the least frequent (5.4%, 2/37). Baseline characteristics showed no significant differences between the cohorts (Table 2).

**Table 2:** Comparison of clinical characteristics between the ECCC sequencing cohort and the ECCC tissue microarray (TMA) cohort.

Parameter	Total	ECCC sequencing cohort	ECCC TMA cohort	p value
	(n = 51)	(n = 35)	(n = 16)	
**Age (years)**				
Median (range)	65 (38–76)	63 (38–76)	66 (51–76)	0.110
**Menopausal status**				
Premenopausal	4 (7.8%)	3 (8.6%)	1 (6.3%)	1.000
Postmenopausal	47 (92.2%)	32 (91.4%)	15 (93.7%)	
**Tumour size (cm)**				
Median (range)	3 (0.7–12)	3 (0.7–12)	3.5 (1.2–8)	0.630
**Metastasis-stratified group**				
Pn	27 (52.9%)	18 (51.4%)	9 (56.2%)	0.749
Pm	24 (47.1%)	17 (48.6%)	7 (43.8%)	
**FIGO 2023 stag**e				
I	3 (5.8%)	3 (8.6%)	0 (0.0%)	0.518
II	26 (51.0%)	18 (51.4%)	8 (50.0%)	
III	21 (41.2%)	13 (37.1%)	8 (50.0%)	
IV	1 (2.0%)	1 (2.9%)	0 (0.0%)	
**Myometrial invasion**				
No or ≤ 1/2	38 (74.5%)	25 (71.4%)	13 (81.3%)	0.730
>1/2	13 (25.5%)	10 (28.6%)	3 (18.7%)	
**LVSI**				
Positive	10 (19.6%)	6 (17.1%)	4 (25.0%)	0.705
Negative	41 (80.4%)	29 (82.9%)	12 (75.0%)	
**ER status**				
Positive	12 (23.5%)	10 (28.6%)	2 (12.5%)	0.296
Negative	39 (76.5%)	25 (71.4%)	14 (87.5%)	
**PR status**				
Positive	4 (7.8%)	3 (8.6%)	1 (6.3%)	1.000
Negative	47 (92.2%)	32 (91.4%)	15 (93.7%)	
**Molecular subtypes**				
POLE-mut	0 (0.0%)	0 (0.0%)	0 (0.0%)	0.723
MMRd	9 (17.7%)	6 (17.1%)	3 (18.8%)	
p53abn	20 (39.2%)	15 (42.9%)	5 (31.2%)	
NSMP	22 (43.1%)	14 (40.0%)	8 (50.0%)	
**Ascites cytology**				
Positive	3 (5.9%)	2 (5.7%)	1 (6.2%)	1.000
Negative	48 (94.1%)	33 (94.3%)	15 (93.8%)	
**Surgical procedure**				
THBSO	2 (3.9%)	2 (5.7%)	0 (0.0%)	1.000
THBSO + PLND/PaLND	49 (96.1%)	33 (94.3%)	16 (100.0%)	
**Adjuvant therapy**				
No therapy	14 (27.4%)	12 (34.3%)	2 (12.5%)	0.145
Radiotherapy alone	2 (3.9%)	2 (5.7%)	0 (0.0%)	
Chemotherapy alone	19 (37.3%)	13 (37.1%)	6 (37.5%)	
Chemoradiotherapy	16 (31.4%)	8 (22.9%)	8 (50.0%)	
**OS**				
Event	16 (31.4%)	12 (34.3%)	4 (25.0%)	0.746
Censored	35 (68.6%)	23 (65.7%)	12 (75.0%)	

Pn, non-metastatic primary tumour; Pm, metastatic primary tumour; LVSI, lymphovascular space invasion; ER, oestrogen receptor; PR, progesterone receptor; POLE-mut, POLE-mutated; MMRd, mismatch repair-deficient; p53abn, p53 abnormal; NSMP, no specific molecular profile; THBSO, total hysterectomy with bilateral salpingo-oophorectomy; PLND, pelvic lymphadenectomy; PaLND, para-aortic lymphadenectomy; OS, overall survival. Continuous variables are reported as mean (range) and analysed using the Mann–Whitney U test. Categorical variables were compared using the Chi-squared test or Fisher's exact test (for expected cell counts <5).

A two-tailed p-value <0.05 was considered statistically significant.

To explore the molecular mechanisms underlying ECCC metastasis, primary tumours were stratified into two groups based on metastatic outcome: Pn (tumours confined to the uterus with no evidence of metastasis during ≥2 years of follow-up) and Pm (metastasis present at initial diagnosis or occurring within 2 years after surgery). The most common metastatic site in the Pm group (n = 24: 17 from the sequencing cohort, 7 from the TMA cohort) was pelvic and/or para-aortic lymph nodes (62.5%, 15/24), followed by pelvic peritoneum (16.7%, 4/24) and lung (12.5%, 3/24) (Fig. 1a). The median follow-up period was 41 months (range: 1–91 months), during which 16 individuals (31.4%, 16/51) died (4 in Pn, 12 in Pm). Clinicopathological analyses revealed that individuals with Pm tumours had a significantly higher proportion of advanced FIGO stages (p < 0.001, chi-square test) and higher mortality rates (p = 0.014, Fisher's exact test; Table 3 and Fig. S1c). Survival analysis demonstrated significantly shorter OS in individuals with Pm tumours (p = 0.004, log-rank test, Fig. 1b). However, no significant difference in OS was observed among individuals with different routes of metastasis (direct extension, haematogenous spread, or lymphatic spread) (p = 0.800, log-rank test; Fig. S1d). Furthermore, adjuvant therapy did not significantly improve OS (p = 0.500, log-rank test, Fig. 1c). This finding may be attributed to limited sample size or the inherent treatment resistance of ECCC. Although molecular subtypes did exhibit significant prognostic stratification value for OS (p = 0.016, log-rank test; Fig. 1d), they were insufficient as independent predictors to effectively differentiate between Pn and Pm tumours (p = 0.662, chi-square test; Table 3 and Fig. S1c).

**Table 3:** Comparison of clinical characteristics of individuals with ECCC stratified by primary tumour metastasis status.

Parameter	Total	Pn	Pm	p value
	(n = 51)	(n = 27)	(n = 24)	
**Cohort**				
ECCC sequencing cohort	35 (68.6%)	18 (66.7%)	17 (70.8%)	0.749
ECCC TMA cohort	16 (31.4%)	9 (33.3%)	7 (29.2%)	
**Age (years)**				
Median (range)	65 (38–76)	64 (49–76)	66 (38–76)	0.219
**Menopausal status**				
Premenopausal	4 (7.8%)	1 (3.7%)	3 (12.5%)	0.331
Postmenopausal	47 (92.2%)	26 (96.3%)	21 (87.5%)	
**Tumour size (cm)**				
Median (range)	3 (0.7–12)	3 (0.7–12)	3.3 (1–8)	0.339
**FIGO 2023 stage**				
I	3 (5.8%)	3 (11.1%)	0 (0.0%)	<0.001
II	26 (51.0%)	23 (85.2%)	3 (12.5%)	
III	21 (41.2%)	1 (3.7%)	20 (83.3%)	
IV	1 (2.0%)	0 (0.0%)	1 (4.2%)	
**Myometrial invasion**				
No or ≤ 1/2	38 (74.5%)	23 (85.2%)	15 (62.5%)	0.107
>1/2	13 (25.5%)	4 (14.8%)	9 (37.5%)	
**LVSI**				
Positive	10 (19.6%)	4 (14.8%)	6 (25.0%)	0.485
Negative	41 (80.4%)	23 (85.2%)	18 (75.0%)	
**ER status**				
Positive	12 (23.5%)	7 (25.9%)	5 (20.8%)	0.669
Negative	39 (76.5%)	20 (74.1%)	19 (79.2%)	
**PR status**				
Positive	4 (7.8%)	3 (11.1%)	1 (4.2%)	0.612
Negative	47 (92.2%)	24 (88.9%)	23 (95.8%)	
**Molecular subtypes**				
POLE-mut	0 (0.0%)	0 (0.0%)	0 (0.0%)	0.662
MMRd	9 (17.7%)	6 (22.3%)	3 (12.5%)	
p53abn	20 (39.2%)	10 (37.0%)	10 (41.7%)	
NSMP	22 (43.1%)	11 (40.7%)	11 (45.8%)	
**Ascites cytology**				
Positive	3 (5.9%)	1 (3.7%)	2 (8.3%)	0.612
Negative	48 (94.1%)	26 (96.3%)	22 (91.7%)	
**Surgical procedure**				
THBSO	2 (3.9%)	2 (7.4%)	0 (0.0%)	0.492
THBSO + PLND/PaLND	49 (96.1%)	25 (92.6%)	24 (100.0%)	
**Adjuvant therapy**				
No therapy	14 (27.4%)	8 (29.6%)	6 (25.0%)	0.490
Radiotherapy alone	2 (3.9%)	0 (0.0%)	2 (8.3%)	
Chemotherapy alone	19 (37.3%)	10 (37.0%)	9 (37.5%)	
Chemoradiotherapy	16 (31.4%)	9 (33.4%)	7 (29.2%)	
**OS**				
Event	16 (31.4%)	4 (14.8%)	12 (50.0%)	0.014
Censored	35 (68.6%)	23 (85.2%)	12 (50.0%)	

Pn, non-metastatic primary tumour; Pm, metastatic primary tumour; LVSI, lymphovascular space invasion; ER, oestrogen receptor; PR, progesterone receptor; POLE-mut, POLE-mutated; MMRd, mismatch repair-deficient; p53abn, p53 abnormal; NSMP, no specific molecular profile; THBSO, total hysterectomy with bilateral salpingo-oophorectomy; PLND, pelvic lymphadenectomy; PaLND, para-aortic lymphadenectomy; OS, overall survival. Continuous variables are reported as mean (range) and analysed using the Mann–Whitney U test. Categorical variables were compared using the Chi-squared test or Fisher's exact test (for expected cell counts <5).

A two-tailed p-value <0.05 was considered statistically significant.

### Transcriptomic profiling reveals expression dysregulation and pathway remodelling in Pm tumours

**Fig. 2:**
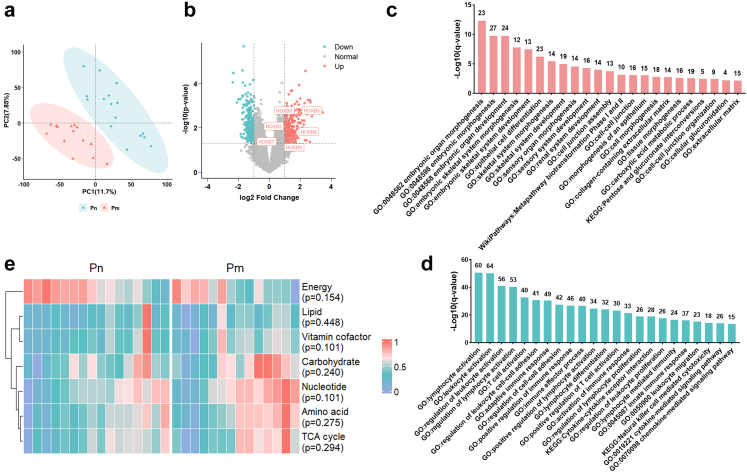
**Transcriptomic profiling reveals expression dysregulation and pathway remodelling in Pm tumours (Pn, n = 16; Pm, n = 14)**. a. PLS-DA of transcriptomic profiles distinguishing Pn and Pm tumours, presented using the first two principal components (PC1: 11.7%; PC2: 7.85%). Each point represents an individual sample, with ellipses denoting the 95% confidence region for each group. b. Volcano plot of DEGs in Pm versus Pn tumours. Axes show log2 FC (x) and -log10 (p-value) (y). Dashed lines indicate thresholds (|log2 FC| ≥ 1; p < 0.05). Points are coloured by differential expression status. p-Values were generated using an empirical Bayes moderated t-test. Selected *HOXB* family members are highlighted. c–d. Functional enrichment analysis of upregulated (c) and downregulated (d) DEGs across the GO, KEGG, and WikiPathways databases. The y-axis shows the enrichment significance (–log10 (q-value)), derived from a hypergeometric test with Benjamini-Hochberg adjustment for multiple comparisons. The x-axis lists significantly enriched terms. Numbers above bars indicate the count of enriched genes per term. e. Heatmap comparing metabolic signatures between Pm and Pn tumours, displaying min–max scaled Z-scores (range 0–1) for selected pathways. p-Values were determined by the Mann–Whitney U test. Pn, non-metastatic primary tumours; Pm, metastatic primary tumours; PLS-DA, partial least squares-discriminant analysis; DEG, differentially expressed gene; log2 FC, log2 Fold Change.

Following stringent quality control of transcriptomic data from the ECCC sequencing cohort (n = 35), five substandard samples were subsequently excluded, retaining 30 high-quality samples (Pn = 16, Pm = 14) for analysis (Fig. 1a). PLS-DA demonstrated significant transcriptomic heterogeneity between Pm and Pn tumours (Fig. 2a). Differential expression analysis identified 453 DEGs, with 218 specifically upregulated and 235 downregulated in the Pm group (Fig. 2b and Supplementary Table S2). Functional enrichment indicated that upregulated DEGs were predominantly involved in embryonic organ development and morphogenesis, epithelial cell differentiation, extracellular matrix organisation, as well as pentose and glucuronate interconversions (Fig. 2c). Notably, *HOXB* family members (*HOXB3*, *HOXB5*, *HOXB6*, *HOXB7*, *HOXB8*, and *HOXB9*) exhibited coordinated overexpression in Pm tumours, suggesting their association with metastatic competence (Fig. 2b). Moreover, Pm tumours demonstrated suppression of immune-related functions, including immune cell activation and regulation, natural killer cell-mediated cytotoxicity, and chemokine-mediated signalling pathways (Fig. 2d).

**Supplementary Figure S2:**
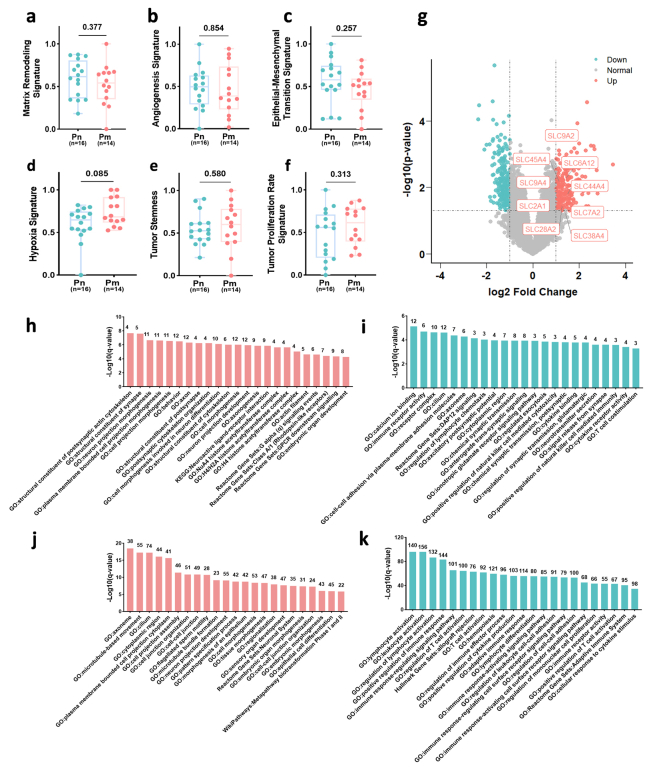
Transcriptomic profiling reveals expression dysregulation and pathway remodelling in Pm tumours (Pn, n = 16; Pm, n = 14), related to Fig. 2 a–f. Comparison of metastasis-associated signature scores between Pm and Pn tumours: matrix remodelling (a, p = 0.377), angiogenesis (b, p = 0.854), epithelial–mesenchymal transition (c, p = 0.257), hypoxia (d, p = 0.085), tumour stemness (e, p = 0.580), proliferation (f, p = 0.313). All p-values were determined by the Mann–Whitney U test. g. Volcano plot of DEGs in Pm versus Pn tumours, highlighting significantly upregulated solute carrier family genes. Axes show log2 FC (x) and –log10 (p-value) (y). Dashed lines indicate thresholds (|log2 FC| ≥ 1; p < 0.05). Points are coloured by differential expression status. p-Values were generated using an empirical Bayes moderated t-test. h–i. Functional enrichment analysis of upregulated (h) and downregulated (i) DEGs in Pm versus Pn tumours within the p53abn subtype across the GO, KEGG, and Reactome databases. The y-axis shows the enrichment significance (–log10(q-value)), derived from a hypergeometric test with Benjamini–Hochberg adjustment. The x-axis lists significantly enriched terms. Numbers above bars indicate the count of enriched genes per term. j–k. Functional enrichment analysis of upregulated (j) and downregulated (k) DEGs within the NSMP subtype across the GO, WikiPathways, Reactome, and Hallmark databases (details as in h–i). Pn, non-metastatic primary tumours; Pm, metastatic primary tumours; DEG, differentially expressed gene; log2 FC, log2 Fold Change; p53abn, p53 abnormal; NSMP, no specific molecular profile.

Comparative analysis of metastasis-associated signatures (matrix remodelling, angiogenesis, epithelial–mesenchymal transition, hypoxia, tumour stemness, and proliferation) revealed no significant differences between Pm and Pn groups (all p > 0.05, Mann–Whitney U test; Fig. S2a–f), indicating that these factors are unlikely to be primary drivers of ECCC metastasis. Solute carrier (*SLC*) family genes, which encode membrane transport proteins associated with metabolism, were significantly upregulated in Pm tumours (Fig. S2g). Concurrently, elevated metabolic activity related to carbohydrates, nucleotides, and amino acids was observed in Pm tumours compared with Pn tumours, although these differences were not statistically significant (Fig. 2e).

To characterise subtype-specific transcriptional features underlying metastasis, and given the limited MMRd samples (n = 3) in the sequencing cohort (Supplementary Table S3), we conducted comparative analyses between Pm and Pn tumours within the p53abn (Pn = 7, Pm = 8) and NSMP (Pn = 7, Pm = 5) subtypes. Functional annotations of DEGs in these subtype-specific comparisons aligned with whole cohort findings (Fig. S2h–k), particularly regarding downregulated DEG enrichment in immune cell activation and immune response regulation pathways. These results underscore the pivotal role of immune dysregulation in shaping metastasis-permissive microenvironments in ECCC.

### The tumour microenvironment (TME) of Pm exhibits immunosuppressive signatures

**Fig. 3:**
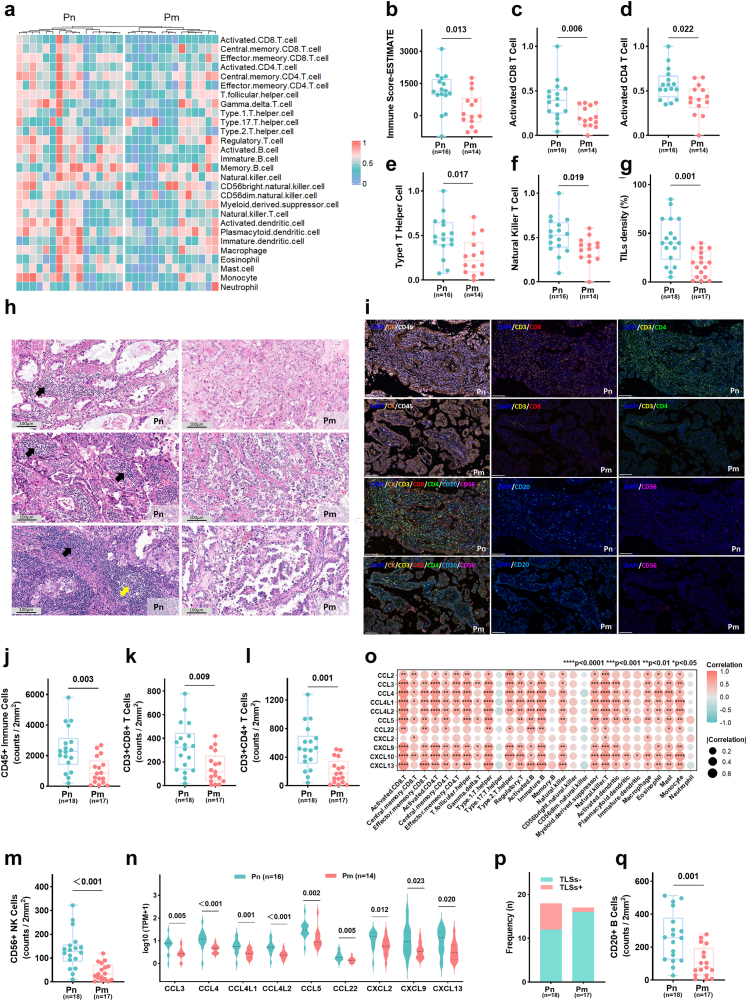
**The TME of Pm exhibits immunosuppressive signatures**. a–f, n–o. Analysis of the ECCC sequencing cohort after quality control of transcriptomic data (Pn, n = 16; Pm, n = 14). a. Heatmap of immune cell infiltration profiles, stratified by metastatic status. Cell enrichment scores were calculated using ssGSEA, min–max scaled Z-scores (range 0–1), and hierarchically clustered with predefined Pn and Pm groups. b. Immune scores, calculated by the ESTIMATE algorithm, compared between Pn and Pm groups (p = 0.013). c–f. Comparison of key anti-tumour immune subset scores between groups: activated CD8 T cells (c, p = 0.006), activated CD4 T cells (d, p = 0.022), Type 1 T helper cells (e, p = 0.017), and natural killer cells (f, p = 0.019). g–h, p–q. Analysis of the entire ECCC sequencing cohort (Pn, n = 18; Pm, n = 17). g. TIL density in Pm versus Pn tumours (p = 0.001). h. Representative haematoxylin and eosin-stained WSI showing TIL infiltration (black arrows) and TLSs (yellow arrows). Scale bar = 100 μm. i. mIF staining in Pm versus Pn tumours: CK^+^ tumour cells (orange), CD45^+^ lymphocytes (white), CD3^+^CD8^+^ T cells (yellow/red), CD3^+^CD4^+^ T cells (yellow/green), CD20^+^ B cells (blue), and CD56^+^ NK cells (magenta). Scale bar = 100 μm. j-m. Quantification of infiltrating immune cells per 2 mm^2^ in Pm versus Pn tumours: CD45^+^ lymphocytes (j, p = 0.003), CD3^+^CD8^+^ T cells (k, p = 0.009), CD3^+^CD4^+^ T cells (l, p = 0.001), and CD56^+^ NK cells (m, p < 0.001). n. Chemokine ligand expression levels in Pm versus Pn tumours (all p < 0.05). o. Correlation between chemokine ligand expression and immune cell enrichment scores. Point colour and size represent the Spearman correlation coefficient. Statistical significance is denoted as follows: ∗p < 0.05, ∗∗p < 0.01, ∗∗∗p < 0.001, ∗∗∗∗p < 0.0001. P-values were derived from Spearman correlation test. p. Frequency of TLSs in Pm versus Pn tumours. q. CD20^+^ B cell quantification per 2 mm^2^ in Pm versus Pn tumours (p = 0.001). All p-values were determined by the Mann–Whitney U test unless otherwise specified. TME, tumour microenvironment; Pn, non-metastatic primary tumours; Pm, metastatic primary tumours; WSI, whole-slide images; ssGSEA, single-sample gene set enrichment analysis; TIL, tumour-infiltrating lymphocyte; TLS, tertiary lymphoid structure; mIF, multiplex immunofluorescence.

**Supplementary Figure S3:**
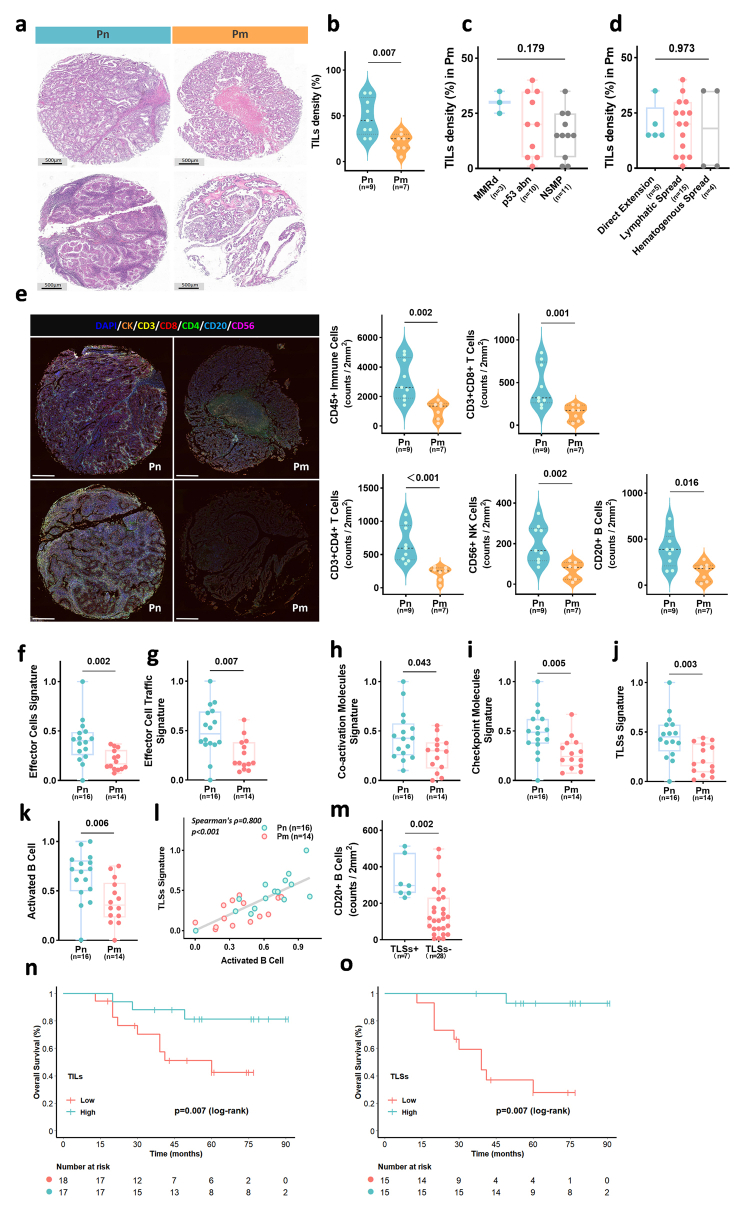
The TME of Pm exhibits immunosuppressive signatures, related to Fig. 3 a. Representative haematoxylin and eosin-stained WSI of the ECCC TMA cohort (n = 16). Scale bar = 500 μm. b. TIL density in Pm versus Pn tumours of the ECCC TMA cohort (n = 16, p = 0.007, Mann–Whitney U test). c. TIL density by molecular subtype in all Pm tumours (n = 24; p = 0.179, Kruskal–Wallis test). d. TIL density by metastatic route in all Pm tumours (n = 24; p = 0.973, Kruskal–Wallis test). e. mIF staining in Pm versus Pn tumours of the ECCC TMA cohort (n = 16), with quantification of infiltrating immune cells per 2 mm^2^: CD45^+^ lymphocytes, CD3^+^CD8^+^ T cells, CD3^+^CD4^+^ T cells, CD56^+^ NK cells, and CD20^+^ B cells (all p < 0.05, Mann–Whitney U test). Scale bar = 500 μm. f–l. Analysis of the ECCC sequencing cohort after quality control of transcriptomic data (Pn, n = 16; Pm, n = 14). f–i, Immune-related signature scores in Pm versus Pn tumours: effector cell signature (f), effector cell traffic signature (g), co-activated molecules signature (h), and immune checkpoint molecule signature (i) (all p < 0.05, Mann–Whitney U test). j. TLS signature scores in Pm versus Pn tumours (p = 0.003, Mann–Whitney U test). k. Activated B cell scores in Pm versus Pn tumours (p = 0.006, Mann–Whitney U test). l. Correlation between activated B cell and TLS signature scores (ρ = 0.800, p < 0.001, Spearman correlation test). m. CD20^+^ B cell quantification per 2 mm^2^ in tumours with versus without TLS in the ECCC sequencing cohort (n = 35, p = 0.002, Mann–Whitney U test). n. Kaplan–Meier analysis of OS by median TIL density in the ECCC sequencing cohort (n = 35; p = 0.007, log-rank test). o. Kaplan–Meier analysis of OS by median TLS signature score in the ECCC sequencing cohort after transcriptomic data quality control (n = 30; p < 0.001, log-rank test). TME, tumour microenvironment; WSI, whole-slide images; ECCC, endometrial clear cell carcinoma; TMA, tissue microarray; TIL, tumour-infiltrating lymphocyte; TLS, tertiary lymphoid structure; Pn, non-metastatic primary tumours; Pm, metastatic primary tumours; mIF, multiplex immunofluorescence; OS, overall survival.

We conducted a comprehensive characterisation of the TME in ECCC, focussing on immune cell infiltration (Fig. 3a). When compared to Pn tumours, Pm tumours exhibited significantly lower immune scores (p = 0.013) and reduced infiltration of key anti-tumour immune subsets: activated CD8 T cells (p = 0.006), activated CD4 T cells (p = 0.022), type 1 helper T cells (p = 0.017), and natural killer (NK) cells (p = 0.019) (all Mann–Whitney U test; Fig. 3b–f). TIL density was decreased in the Pm group across both sequencing (p = 0.001, Mann–Whitney U test; Fig. 3g–h) and TMA cohorts (p = 0.007, Mann–Whitney U test; Fig. S3a–b). Reduced TIL density was independent of molecular subtypes (p = 0.179, Kruskal–Wallis test; Fig. S3c) and metastatic route (p = 0.973, Kruskal–Wallis test; Fig. S3d), indicating a pervasive immune-cold phenotype in primary tumours of metastatic ECCC, potentially driven by tumour-intrinsic immunoediting.

mIF assays in two independent cohorts confirmed reduced infiltration of CD45^+^ lymphocytes, CD3^+^CD8^+^ T cells, CD3^+^CD4^+^ T cells, and CD56^+^ NK cells in Pm compared to Pn tumours (all p < 0.05, Mann–Whitney U test; Fig. 3i–m and Fig. S3e). Decreased chemokine ligand expression in Pm tumours (Fig. 3n) positively correlated with immune cell abundance (Fig. 3o), suggesting impaired immune recruitment. Consistent with these findings, Pm tumours exhibited lower effector cell function (p = 0.002, Mann–Whitney U test; Fig. S3f) and trafficking signature scores (p = 0.007, Mann–Whitney U test; Fig. S3g). Concurrent downregulation of co-stimulatory (p = 0.043, Mann–Whitney U test; Fig. S3h) and immune checkpoint signatures (p = 0.005, Mann–Whitney U test; Fig. S3i) was observed, potentially diminishing immunotherapeutic responses.

TLSs were more frequent in Pn tumours (Fig. 3h and p), exhibiting higher TLS signature scores than Pm tumours (p = 0.003, Mann–Whitney U test; Fig. S3j). As critical immune hubs, TLS maturation is associated with B cell activity.^39^^,^^40^ Increased TLSs in Pn tumours correlated with elevated activated B cell abundance (p = 0.006, Mann–Whitney U test; Fig. S3k), showing a strong correlation between activated B cell abundance and TLS signature scores (ρ = 0.800, p < 0.001, Spearman correlation; Fig. S3l). mIF confirmed higher CD20^+^ B cell infiltration in TLS^+^ versus TLS^−^ lesions (p = 0.002, Mann–Whitney U test; Fig. S3m), with consistently greater CD20^+^ B cell infiltration in Pn tumours across both cohorts (all p < 0.05, Mann–Whitney U test; Fig. 3q and Fig. S3e). Higher TIL density (p = 0.007, log-rank test; Fig. S3n) and TLS signature scores (p < 0.001, log-rank test; Fig. S3o) correlated with prolonged survival. Collectively, Pm tumours display reduced anti-tumour immunity, impaired chemokine-mediated recruitment, and compromised TLS formation, fostering an immunosuppressive TME.

### Genome-wide DNA methylation profiling reveals metastasis-specific epigenomic remodelling

**Fig. 4:**
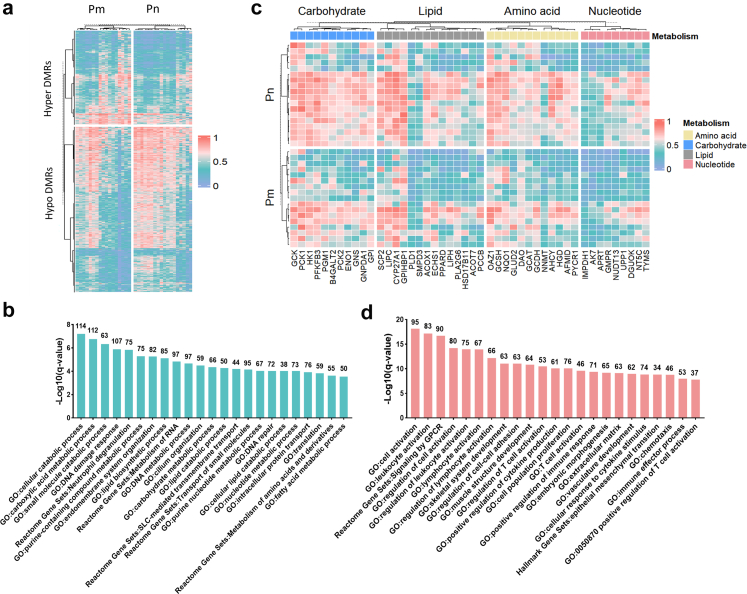
**Genome-wide DNA methylation profiling reveals metastasis-specific epigenomic remodelling (Pn, n = 18; Pm, n = 17)**. a. Heatmap of promoter DMRs, showing 2907 hypomethylated and 1717 hypermethylated DMRs in Pm versus Pn tumours. Methylation β-values (range 0–1) were hierarchically clustered with predefined groups. b. Functional enrichment analysis of hypomethylated DMGs in promoter regions across the GO and Reactome databases. The y-axis shows the enrichment significance (–log10 (q-value)), and the x-axis lists significantly enriched terms. Numbers above bars indicate the count of enriched genes per term. c. Heatmap of methylation levels for metabolic pathway genes, stratified by metastatic status. Methylation β-values (range 0–1) were hierarchically clustered with predefined groups. Colour bars denote distinct metabolic pathways. d. Functional enrichment analysis of hypermethylated DMGs in promoter regions across the GO, Reactome, and Hallmark databases (details as in b). DMR, differentially methylated region; DMG, differentially methylated gene; Pn, non-metastatic primary tumours; Pm, metastatic primary tumours.

**Supplementary Figure S4:**
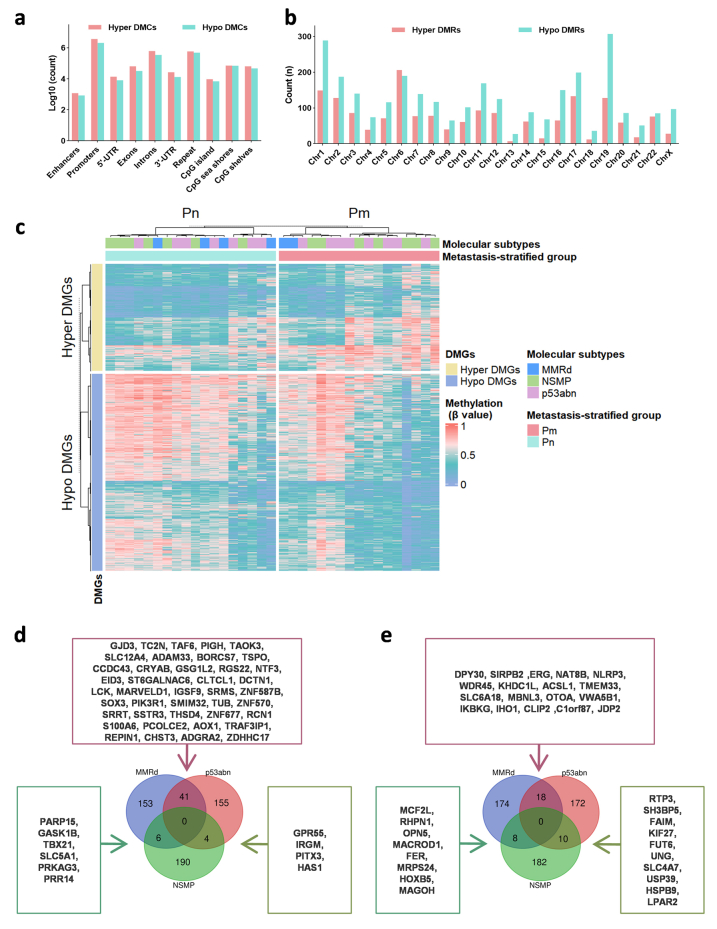
Genome-wide DNA methylation profiling reveals metastasis-specific epigenomic remodelling (Pn, n = 18; Pm, n = 17), related to Fig. 4 a. Genomic distribution of DMCs identified in Pm versus Pn tumours. The x-axis represents genomic features; the y-axis shows the log10-transformed count of DMCs. b. Chromosomal distribution of promoter DMRs in Pm versus Pn tumours. The x-axis lists chromosomes; the y-axis indicates the count of DMRs. c. Heatmap of promoter DMGs in Pm versus Pn tumours, stratified by molecular subtype. Methylation β-values (range 0–1) were hierarchically clustered with predefined groups. d–e. Overlap analysis of molecular subtype-specific DMGs: Venn diagrams showing concordance of the top 200 hypermethylated (d) and hypomethylated (e) DMGs across subtypes; numbers indicate overlapping gene counts. DMC, differentially methylated cytosine; DMR, differentially methylated region; DMG, differentially methylated gene; Pn, non-metastatic primary tumours; Pm, metastatic primary tumours; MMRd, mismatch repair deficient; p53abn, p53 abnormal; NSMP, no specific molecular profile.

Methylation analysis of the sequencing cohort (17 Pm versus 18 Pn tumours) identified 2,072,367 DMCs, comprising 40.5% hypomethylated and 59.5% hypermethylated sites, with the maximum aberrant methylation frequency observed in promoter regions (Fig. S4a). We detected 79,143 DMRs, including 2907 hypomethylated and 1717 hypermethylated DMRs in promoters (Fig. 4a and Supplementary Table S4). Hypomethylation events exhibited a broader distribution in Pm tumours, showing particular enrichment on chromosomes 1, 19, and X (Fig. S4b). Functional enrichment of promoter-associated differentially methylated genes (DMGs) defined by DMR-neighbour mapping revealed 2232 hypomethylated DMGs significantly involved in metabolic processes (e.g. carbohydrate metabolism, lipid catabolism, nucleotide metabolism, amino acid and derivative metabolism), DNA damage response, *SLC*-mediated transmembrane transport, and intracellular protein transport (Fig. 4b). Concordantly, genes encoding metabolic rate-limiting enzymes (e.g. *GCK*, *ENO1*, *HK1*) displayed promoter hypomethylation in Pm tumours (Fig. 4c), indicating that DNA hypomethylation-driven metabolic reprogramming sustains biosynthetic activity to fuel proliferation and metastasis. Furthermore, 1253 hypermethylated DMGs were enriched in immune pathways, including immune cell activation and regulation (e.g. lymphocyte activation, T cell activation, regulation of T cell activation), immune effector processes, cell–cell adhesion regulation, and cellular responses to cytokine stimuli (Fig. 4d), suggesting that DNA hypermethylation shapes an immunosuppressive microenvironment through silencing immune-activating genes. Importantly, these metastasis-associated epigenetic alterations demonstrated prevalence across all molecular subtypes without a dependency on specific subtypes (Fig. S4c). Comparative analysis of the top 200 most significantly altered DMGs per molecular subtype revealed no common DMGs across all three subtypes, partial overlap between MMRd and p53abn subtypes (41 hypermethylated and 18 hypomethylated DMGs), indicating shared epigenetic alterations, and minimal overlap with pronounced heterogeneity in NSMP tumours (Fig. S4d–e).

### Epigenetic silencing of ETS1 regulon compromises anti-tumour immunity

**Fig. 5:**
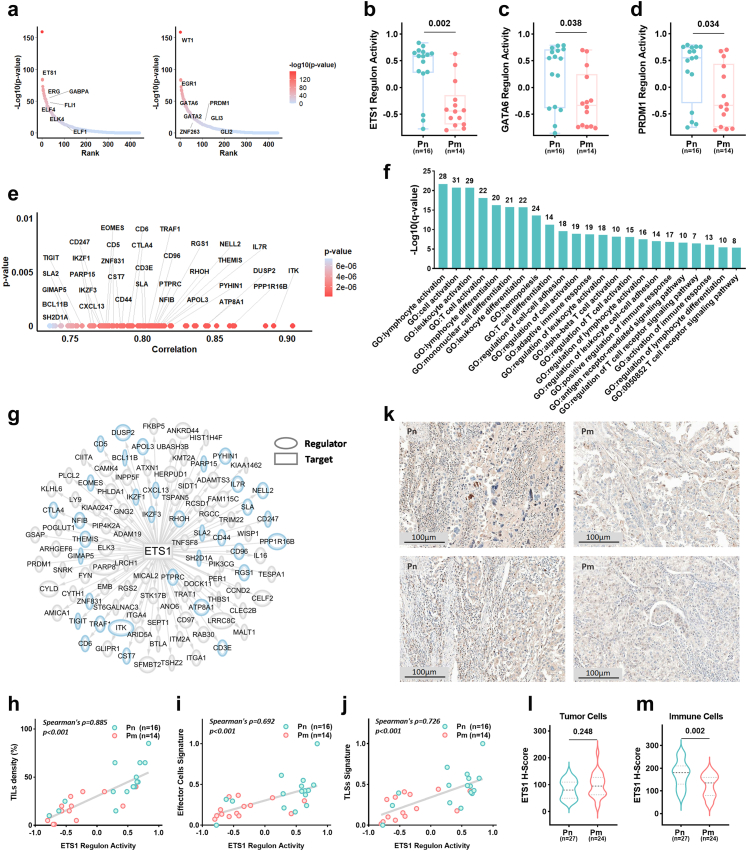
**Epigenetic silencing of the ETS1 regulon compromises anti-tumour immunity**. a–g. Analysis of the ECCC sequencing cohort with paired transcriptomic and methylation data (Pn, n = 16; Pm, n = 14). a. Motif enrichment analysis of immune-related hypermethylated DMRs. The y-axis shows the enrichment significance (–log10 (p-value)); the x-axis indicates the motif rank. Significant enrichment was observed for transcription factor binding motifs from the ETS (left) and ZF (right) families. p-Values were determined using a hypergeometric test. b–d. Reduced regulon activity of ETS1 (b, p = 0.002), GATA6 (c, p = 0.038), and PRDM1 (d, p = 0.034) in Pm versus Pn tumours. e. Correlation between ETS1 expression and its target genes. The x-axis represents the Spearman correlation coefficient; the y-axis shows the corresponding p-value. p-Values were calculated by Spearman correlation test. f. Functional enrichment analysis of ETS1 target genes across the GO database. The y-axis shows the enrichment significance (–log10 (q-value)), derived from a hypergeometric test with Benjamini–Hochberg adjustment. The x-axis lists significantly enriched terms. Numbers above bars indicate the count of enriched genes per term. g. Regulatory network of the ETS1 regulon. Transcription factors are represented as ovals and target genes as rectangles; connecting lines indicate regulatory interactions. Oval size corresponds to interaction strength; blue borders denote targets overlapping with downregulated DEGs. h–j. Correlation of ETS1 regulon activity with TME features: TIL density (h, ρ = 0.885, p < 0.001), effector cell signature score (i, ρ = 0.692, p < 0.001), and TLS signature score (j, ρ = 0.726, p < 0.001). p-Values were calculated by Spearman correlation test. k–m, Analyses were performed on the overall ECCC cohort (Pn, n = 27; Pm, n = 24). k. Representative immunohistochemical staining of ETS1. Scale bar = 100 μm. l-m. ETS1 protein expression (H-score) in tumour cells (l, p = 0.248) and immune cells (m, p = 0.002) in Pm versus Pn tumours. All p-values were determined using the Mann–Whitney U test unless otherwise specified. ECCC, endometrial clear cell carcinoma; DMR, differentially methylated region; DMG, differentially methylated gene; Pn, non-metastatic primary tumours; Pm, metastatic primary tumours; DEG, differentially expressed gene; TME, tumour microenvironment; TIL, tumour-infiltrating lymphocyte; TLS, tertiary lymphoid structure.

**Supplementary Figure S5:**
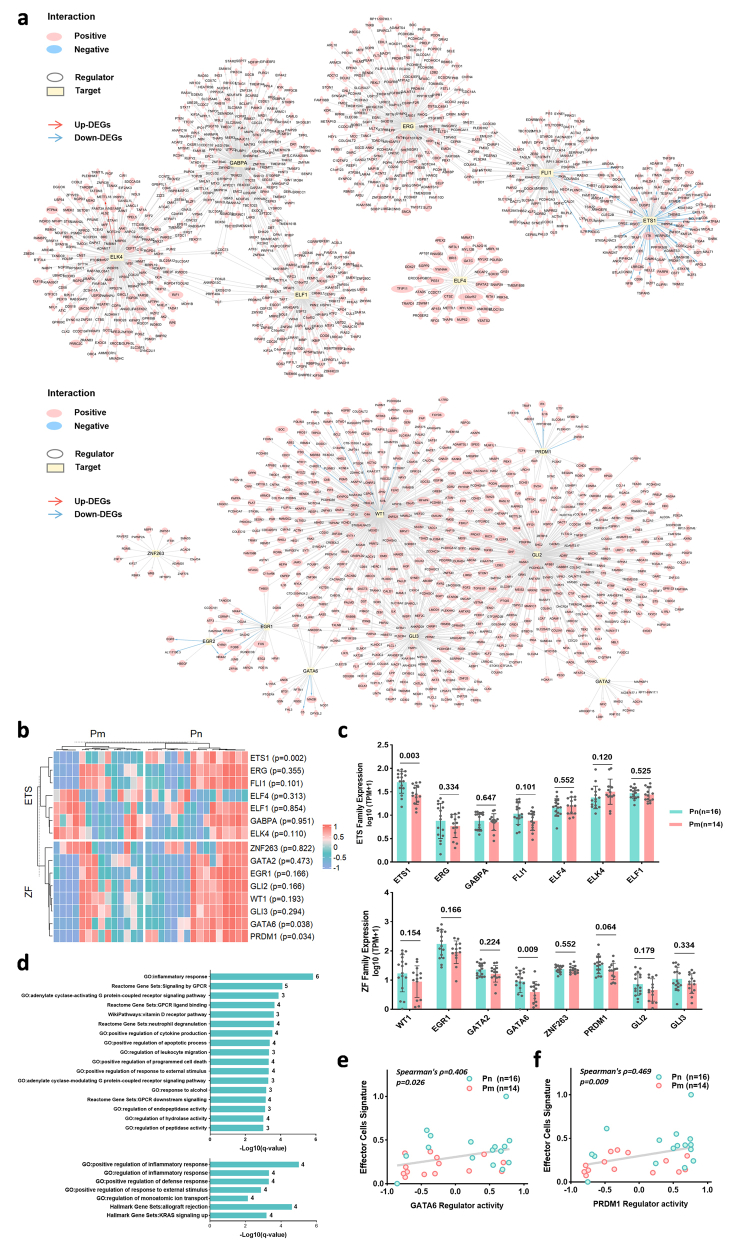
Epigenetic silencing of ETS1 regulon compromises anti-tumour immunity, related to Fig. 5. Analysis of the ECCC sequencing cohort with paired transcriptomic and methylation data (Pn, n = 16; Pm, n = 14). a. Transcriptional regulatory network of ETS (top) and ZF (bottom) family transcription factors. Node shapes distinguish transcription factors (ellipses) from target genes (rectangles); ellipse area reflects interaction strength, and node colour denotes interaction type (positive: red; negative: blue). Arrows mark targets overlapping with DEGs (upregulated: red; downregulated: blue). b. Heatmap of ETS and ZF family regulon activity stratified by metastatic status. Regulon activity scores were derived from GSEA, normalised to the range of–1 to 1, and hierarchically clustered with predefined groups. p-Values were calculated using the Mann–Whitney U test. c. Expression levels of ETS (top) and ZF (bottom) family transcription factors in Pm versus Pn tumours. All p-values were determined by the Mann–Whitney U test. d. Functional enrichment of GATA6 (top) and PRDM1 (bottom) regulon target genes across the GO, WikiPathways, Reactome, and Hallmark databases. The x-axis shows the enrichment significance (–log10(q-value)), derived from a hypergeometric test with Benjamini-Hochberg adjustment. The y-axis lists significantly enriched terms. Numbers above bars indicate the count of enriched genes per term. e–f. Correlation between regulon activity and effector cell signature scores: GATA6 regulon (e, ρ = 0.406, p = 0.026) and PRDM1 regulon (f, ρ = 0.469, p = 0.009), respectively. All p-values were calculated by Spearman correlation test. ECCC, endometrial clear cell carcinoma; Pn, non-metastatic primary tumours; Pm, metastatic primary tumours; DEG, differentially expressed gene; GSEA, gene set enrichment analysis.

Integrated epigenomic-transcriptomic analysis revealed that impaired immune responses in primary tumours of ECCC represent a key immune evasion mechanism independent of molecular subtype. To elucidate the regulatory role of DNA methylation, we analysed its impact on transcription factor (TF) binding sites. Motif analysis of immune-related hypermethylated DMRs showed significant enrichment for ETS and zinc finger (ZF) family TF binding sites (Fig. 5a and Supplementary Table S5), implicating their involvement in methylation-mediated immune evasion. Transcriptional regulatory network analysis identified a positive regulatory network comprising seven ETS family members (ETS1, ERG, GABPA, FLI1, ELF4, ELK6, ELF1) and eight ZF family members (WT1, EGR1, GATA6, GATA2, ZNF263, PRDM1, GLI3, GLI2) (Fig. S5a–b). Notably, Pm tumours exhibited significantly reduced regulon activities of ETS1, GATA6, and PRDM1 (all p < 0.05, Mann–Whitney U test; Fig. 5b–d), with concomitant downregulation of ETS1 and GATA6 expression (all p < 0.05, Mann–Whitney U test; Fig. S5c), indicating dual transcriptional-epigenetic suppression.

To evaluate the impact of ETS1, GATA6, and PRDM1 regulons on immune-related processes, functional dissection was performed. ETS1 positively regulated 103 target genes (Fig. 5e), which were significantly enriched in lymphocyte activation and differentiation, T cell activation and differentiation, as well as immune response activation pathways (Fig. 5f). Notably, 34 of these targets overlapped with immune-related downregulated DEGs (Fig. 5g). Strong positive correlations were observed between ETS1 regulon activity and key immune features: TIL density (ρ = 0.885, p < 0.001), effector cell signature scores (ρ = 0.692, p < 0.001), and TLS signature scores (ρ = 0.726, p < 0.001) (all Spearman correlation, Fig. 5h–j). In contrast, GATA6 (20 targets) and PRDM1 (26 targets) regulons primarily regulated inflammatory responses (Fig. S5d), showing weaker correlations with effector cell signature scores (GATA6: ρ = 0.406, p = 0.026; PRDM1: ρ = 0.469, p = 0.009; Spearman correlation, Fig. S5e–f), highlighting ETS1 as the central immune regulator. IHC confirmed significantly reduced ETS1 protein expression (H-score) in immune cells within the TME of Pm compared to Pn tumours (Fig. 5k-m), with no difference in tumour cells, indicating that ETS1 functionality is primarily localised to immune cells. Collectively, DNA hypermethylation attenuates anti-tumour immunity by modifying ETS1 binding sites, impairing its regulon activity.

### T cell receptor signalling gene hypermethylation drives immunosuppression and predicts metastasis in ECCC

**Fig. 6:**
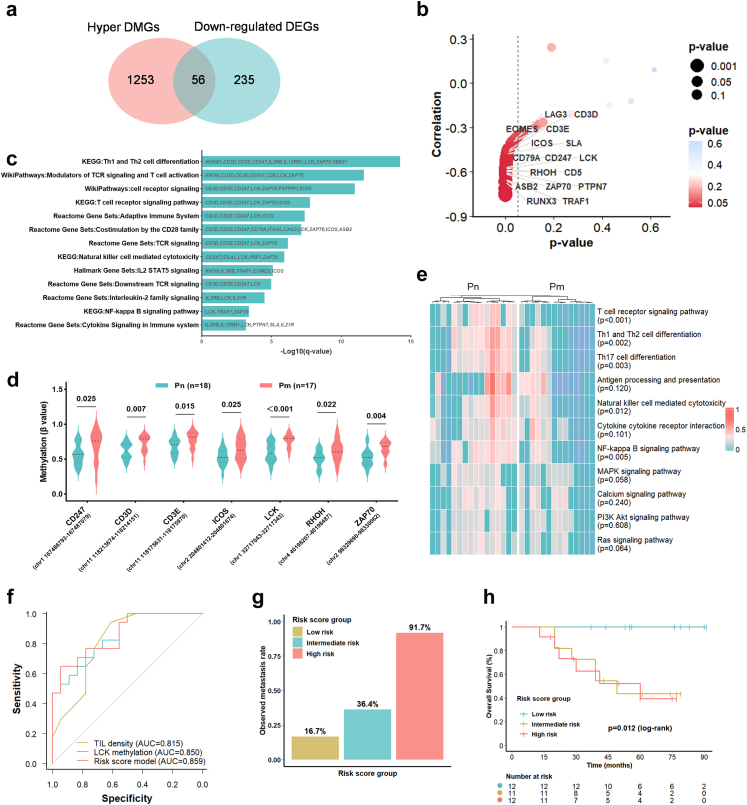
**TCR signalling gene hypermethylation drives immunosuppression and predicts metastasis in ECCC**. a–c, e. Analysis of the ECCC sequencing cohort with paired transcriptomic and methylation data (Pn, n = 16; Pm, n = 14). a. Venn diagram showing the overlap between hypermethylated DMGs and downregulated DEGs; numbers indicate gene counts. b. Methylation-expression correlation for the 56 overlapping genes, showing the Spearman correlation coefficient (y-axis) against the corresponding p-value (x-axis). Point size and colour denote statistical significance. The dashed line indicates the p = 0.05 threshold. p-Values were calculated by Spearman correlation test. c. Functional enrichment analysis of the 43 hypermethylated-downregulated genes across the GO, KEGG, WikiPathways, Reactome, and Hallmark databases. The x-axis shows the enrichment significance (–log10 (q-value)), derived from a hypergeometric test with Benjamini–Hochberg adjustment. The y-axis lists significantly enriched terms. d, f–h, Analysis of the ECCC sequencing cohort with methylation data (Pn, n = 18; Pm, n = 17). d. Differential methylation of TCR signalling pathway genes in Pm versus Pn tumours: *CD247* (p = 0.025), *CD3D* (p = 0.007), *CD3E* (p = 0.015), *ICOS* (p = 0.025), *LCK* (p < 0.001), *RHOH* (p = 0.022), and *ZAP70* (p = 0.004). All p-values were determined using the Mann–Whitney U test. e. Heatmap of activity scores for TCR signalling and interacting immune pathways. Activity scores were calculated from transcriptomic data using GSVA, min–max scaled Z-scores (range 0–1), and hierarchically clustered with predefined groups. f. ROC curves comparing the performance of metastasis prediction metrics: TIL density (AUC = 0.815), *LCK* methylation level (AUC = 0.850), and a combined risk score model (AUC = 0.859). The risk score model showed improved predictive performance over individual metrics. g. Observed metastasis rate in individuals with ECCC, stratified into tertiles by the risk score model (Low risk: 16.7%; Intermediate risk: 36.4%; High risk: 91.7%). The y-axis shows the proportion of individuals with metastatic events. h. Kaplan–Meier analysis of OS stratified by risk score group (Low risk, n = 12; Intermediate risk, n = 11; High risk, n = 12; p = 0.012, log-rank test). TCR, T cell receptor; ECCC, endometrial clear cell carcinoma; DMG, differentially methylated gene; DEG, differentially expressed gene; Pn, non-metastatic primary tumours; Pm, metastatic primary tumours; GSVA, gene set variation analysis; ROC, receiver operating characteristic; AUC, area under the curve; OS, overall survival.

**Supplementary Figure S6:**
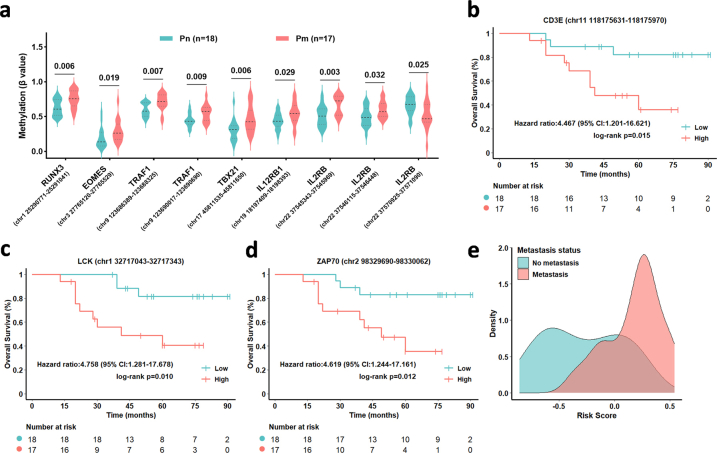
TCR signalling gene hypermethylation drives immunosuppression and predicts metastasis in ECCC (ECCC sequencing cohort; Pn, n = 18; Pm, n = 17), related to Fig. 6 a. DMR methylation levels of immune-related genes in Pm versus Pn tumours (all p < 0.05, Mann–Whitney U test). b–d. Kaplan–Meier analysis of OS stratified by median methylation level of *CD3E* (b, p = 0.015), *LCK* (c, p = 0.010), and *ZAP70* (d, p = 0.012). All p-values were determined by the log-rank test. Hazard ratios and 95% confidence intervals were derived from univariable Cox proportional hazards regression. e. Distribution of risk score by metastasis status. The x-axis represents the computed risk score; the y-axis shows the probability density. Kernel density estimation curves are shown for Pn and Pm groups, illustrating the shift towards higher risk scores in metastatic tumours. TCR, T cell receptor; ECCC, endometrial clear cell carcinoma; DMR, differentially methylated region; Pn, non-metastatic primary tumours; Pm, metastatic primary tumours; OS, overall survival.

DNA hypermethylation in gene promoters typically leads to transcriptional silencing. To identify metastasis-associated immune driver methylation events, methylation-expression correlation analysis was performed on 56 genes exhibiting promoter hypermethylation and downregulated expression in Pm tumours (Fig. 6a–b). Methylation levels of 43 genes showed significant negative correlations with expression (Fig. 6b and Supplementary Table S6), enriched in TCR signalling, Th1 and Th2 cell differentiation, costimulation by the CD28 family, NK cell-mediated cytotoxicity, and NF-κB signalling pathways (Fig. 6c). Strikingly, 11 genes (*RHOH*, *CD5*, *CD3E*, *CD6*, *CD247*, *TRAF1*, *SLA*, *APOL3*, *TIGIT*, *PARP15*, *EOMES*) belonged to the ETS1 regulon. As TCR signalling is critical for T cell immunity,^41^ key pathway genes exhibited significantly elevated methylation in Pm tumours, including TCR-CD3 complex components (*CD247* [p = 0.025], *CD3D* [p = 0.007], *CD3E* [p = 0.015]) and signalling-associated genes (*LCK* [p < 0.001], *ZAP70* [p = 0.004], *ICOS* [p = 0.025], *RHOH* [p = 0.022]) (all Mann–Whitney U test; Fig. 6d). Pathway activity analysis confirmed significantly reduced TCR signalling activity in Pm tumours (p < 0.001), with concomitant decreases in Th1 and Th2 cell differentiation (p = 0.002), Th17 cell differentiation (p = 0.003), NK cell-mediated cytotoxicity (p = 0.012), and NF-κB signalling pathway activity (p = 0.005) (all Mann–Whitney U test, Fig. 6e), indicating that hypermethylation-mediated TCR suppression critically impairs T cell function. Other immune genes (*RUNX3*, *IL2RB*, *IL12RB1*, *TBX21*, *EOMES*, *TRAF1*) also displayed higher methylation in Pm tumours (all p < 0.05, Mann–Whitney U test; Fig. S6a). Collectively, coordinated methylation-expression changes in immune genes represent key epigenetic drivers that shape the immunosuppressive metastatic microenvironment.

To evaluate the potential clinical predictive value of these immune driver genes and avoid overfitting due to limited sample size, we conducted univariable logistic regression. This analysis identified TIL density and the methylation levels of *CD3E*, *LCK*, and *ZAP70* as significant predictors of metastasis (Supplementary Table S7). Hypermethylation of these genes was associated with poorer OS (Fig. S6b–d). LASSO regression, utilising five-fold cross-validation (optimal λ.1se = 0.2025), established a risk score model defined as follows: Risk score = −1.5487 + (−0.0028 × TIL density) + (2.3239 × LCK β-value). This model effectively discriminated between metastasis statuses (Fig. S6e), achieving an AUC of 0.859 (Fig. 6f), thereby outperforming individual predictors. Stratification by risk score tertiles revealed that 91.7% of high-risk patients had metastasis, compared to 36.4% in the intermediate-risk group and 16.7% in the low-risk group (Fig. 6g). The intermediate and high-risk groups demonstrated significantly shorter OS than low-risk patients (p = 0.012, log-rank test; Fig. 6h), highlighting an urgent need for intensified intervention. We emphasise, nonetheless, that this exploratory model requires validation in prospective cohorts.

## Discussion

As a rare yet highly aggressive malignancy, ECCC presents substantial clinical challenges due to the poorly understood molecular mechanisms driving its metastatic progression, coupled with the lack of targeted therapeutic strategies. By integrating multi-omics data, this study systematically characterises the metastasis-specific molecular features of ECCC. We elucidated the core regulatory role of the “epigenetic-immune axis” in driving ECCC metastasis, developed a metastasis risk score model, and identified immune-related epigenetic targets. These findings provide critical theoretical foundations and practical tools for precise risk stratification and personalised management of individuals with ECCC.

Molecular subtypes are integral to clinical management in endometrial cancer, and their utility extends to ECCC, where they demonstrate significant prognostic value. In our cohort, individuals with MMRd exhibited markedly longer survival than those with the NSMP or p53abn subtypes. Notably, no cases of POLE-mut were detected, aligning with the reported low prevalence (2%) of this subtype in ECCC.^8^ The NSMP subtype was predominant (43.1%, 22/51) and displayed epigenetic and transcriptional heterogeneity in our study. Real-world data indicate a higher proportion of NSMP in Chinese cohorts compared to published data on endometrial cancer in white populations.^42^ Similarly, the prevalence of the MMRd subtype was higher in Italian ECCC cohorts (33.3%)^43^ than in our study cohort (17.7%). These cross-population disparities, alongside reports of racial variations in DNA methylation patterns in endometrial cancer,^44^ underscore the role of sociocultural and environmental factors in shaping tumour molecular profiles. Importantly, such findings highlight the need for caution when extrapolating molecular findings across racially diverse populations, as unmeasured confounders (e.g. healthcare access, screening practices, or lifestyle factors) may drive observed differences.

We identified an immunosuppressive TME as a critical feature underlying ECCC metastasis. Primary tumours from individuals who developed metastasis universally exhibited an immune-cold phenotype, characterised by reduced TIL density, impaired immune cell recruitment, and defective TLS formation—particularly diminished mature TLSs. Crucially, this immunosuppressive signature existed independently of molecular subtypes. This observation challenges the traditional view that POLE-mut and MMRd tumours exhibit high immunogenicity due to elevated mutational burden and neoantigen exposure.^7^^,^^8^ It implies that ECCC metastasis may rely more heavily on epigenetically regulated immunosuppressive mechanisms than solely on mutational burden, providing a key rationale for interventions aimed at reversing immunosuppression and enhancing immunotherapy sensitivity.

TLSs, which are lymph node-like functional immune structures within the TME, are associated with favourable prognoses and responses to immunotherapy in various solid tumours.^45^ Our findings suggest that mature TLSs and their core components, B cells, serve as potential protective immune factors against ECCC metastasis. The formation and function of TLSs critically depend on B cell recruitment and activation.^40^ B cells enhance anti-tumour T cell responses through mechanisms including antigen presentation, antibody secretion, and follicular helper T cell activation.^46^ In subjects with melanoma receiving neoadjuvant ICB, responders demonstrated significantly higher intratumoural B cell signature gene expression and TLS density compared to non-responders.^47^ Similar findings in lung adenocarcinoma indicate that B cell-associated TLS signatures independently predict long-term survival benefits from ICB.^48^ These findings support the synergistic role of TLSs and B cells in augmenting anti-tumour immunity. Consequently, strategies combining ICB with targeted B cell activation or TLS induction hold significant promise for improving immunotherapy responses in ECCC.

Our key finding is the “epigenetic-immune axis” mechanism that underlies ECCC metastasis. DNA methylation targets ETS1-binding sites and key genes in the TCR signalling pathway (e.g. *LCK*, *CD3E*, *ZAP70*), systematically silencing immune response genes and reshaping the immunosuppressive TME, thereby enabling metastasis. ETS1, a transcription factor highly expressed in immune cells, regulates lymphocyte activation, differentiation, and function; its knockout impairs T cell proliferation and increases spontaneous apoptosis.^49^ The TCR signalling pathway, critical for T cell activation,^50^ is directly suppressed by the epigenetic silencing of its key genes, thereby weakening anti-tumour immunity. These findings resonate with multiple investigations: Shang et al. analysed The Cancer Genome Atlas (TCGA) endometrial cancer data and linked DNA methylation-mediated silencing of *CIRBP* and *INPP5K* to reduced CD4/CD8 T cell infiltration and poor prognosis^51^; Liu et al. developed a prognostic index (MDS) based on aberrantly methylated driver genes (*PARVG*, *SYNE4*, and *CDO1*), which enables effective survival stratification and reflects tumour immune status in endometrial cancer.^52^ Collectively, these studies provide robust evidence that DNA methylation regulates immune dynamics and informs prognosis in endometrial cancer. Additionally, non-coding RNAs and other epigenetic regulators further expand the toolkit for risk stratification,^53^^,^^54^ underscoring the broad relevance of epigenetic mechanisms in influencing endometrial cancer immunity.

While immunotherapy remains a first-line option for advanced and recurrent endometrial cancer, its efficacy is limited in immune-cold tumours such as ECCC. The potential of epigenetic therapies to dynamically modulate tumour immunogenicity and immune cell function offers a promising avenue for precision oncology.^55^ DNA methyltransferase inhibitors (DNMTis), such as 5-Azacytidine, can reverse abnormal hypermethylation, reactivating silenced tumour antigens and antigen-presentation genes to remodel immunogenicity.^56^^,^^57^ In vivo studies confirm that combining DNMTis with ICB enhances T cell renewal and boosts CD8^+^ T cell anti-tumour activity.^58^^,^^59^ Other epigenetic drugs targeting histone modifications, chromatin remodelling, and non-coding RNAs also demonstrate immune-sensitising potential.^60^ For instance, enhancer of zeste homologue 2 (EZH2) inhibitors augment NK cell cytotoxicity by inhibiting H3K27 methylation,^61^ while pre-treating T cells with histone deacetylase (HDAC) inhibitors significantly enhances their anti-tumour effects in chimeric antigen receptor-engineered T (CAR-T) cell therapy.^62^ Emerging approaches, such as nanoparticle-based targeted delivery and epigenetic editing technologies like CRISPR-dCas9-TET1, promise advancements by allowing precise reversal of specific methylation targets.^63^^,^^64^ Clinical trials are currently assessing the combined use of DNMTis with pembrolizumab in advanced solid tumours (NCT02959437), providing a translational reference for ECCC. However, it is crucial to note that although DNMTis mechanistically target DNA methyltransferases, their genome-wide demethylating action could lead to non-specific biological effects or tissue toxicity, warranting careful safety evaluations in clinical applications.

From the perspective of precision medicine in clinical practice, these advancements not only provide critical candidate strategies for combination therapies in high-risk ECCC patients but also drive the establishment of personalised treatment paradigms. The clinical translation of epigenetic therapies entails multifaceted systemic challenges: First, at the healthcare system level, standardised molecular testing protocols must be implemented to enable accurate patient stratification, necessitating optimisation of current healthcare resource allocation. Second, the unique characteristics of epigenetic therapies—such as dynamic modifications and delayed effects—impose emerging requirements for updating evidence-based clinical guidelines. Achieving these goals demands enhanced interdisciplinary collaboration. For instance: (1) Integration between pathology and bioinformatics can accelerate the development of AI-driven methylation profiling tools (while simultaneously establishing robust frameworks for data privacy protection). (2) Coordination among medical administration departments and ethics committees can ensure informed consent processes adequately address the distinctive risk–benefit profiles of epigenetic interventions. These include the potential for sustained anti-tumour responses (a major advantage, as epigenetic changes may persist after treatment cessation) alongside risks such as off-target epigenetic alterations, which could inadvertently activate oncogenes or silence tumour suppressor genes. These systemic transformations will enhance precision therapy for ECCC and reflect the evolution towards a “dynamic precision” oncology model that accounts for temporal epigenetic modifications.

Overall, this study has established a comprehensive epigenetic framework for the rare malignancy of ECCC, systematically characterising metastasis-specific molecular features and elucidating the “epigenetic-immune axis” as a core driver of metastasis. The developed metastasis risk score model provides a clinically applicable tool for the early identification of high-risk individuals, while discoveries of key epigenetic targets lay the groundwork for the combination of epigenetic therapies and immunotherapy. Limitations of this study include the small cohort size, which necessitates the validation of the risk score model and epigenetic markers in larger prospective studies. Further constrained by the rarity of ECCC and limited access to fresh tissue or well-established cell lines, mechanistic investigations were primarily limited to multi-omics correlation and preliminary in vitro validation, leaving gaps in follow-up in vivo studies and targeted therapy exploration. Finally, dynamic DNA methylation changes across metastatic stages and spatial heterogeneity within the TME require further elucidation. Notwithstanding these limitations, in contrast to previous studies focussing on the clinical implications of epigenetic alterations in a limited set of hotspot genes (e.g. *MLH1*) in ECCC, this genome-wide study systematically elucidates the epigenetic molecular mechanisms by which the immunosuppressive TME drives ECCC metastasis and defines the functional roles of key regulatory nodes. These findings address the gap in research on the epigenetic mechanisms underlying ECCC metastasis, while supporting the development of treatment strategies for ECCC that centre on “epigenetic intervention synergised with immune microenvironment remodelling”. Future advancements will necessitate multicentre collaboration, multidimensional data integration, and rigorous preclinical validation to accelerate clinical translation, thereby advancing precision medicine approaches for individuals with ECCC.

## Contributors

Huiqing Jia: Conceptualisation, investigation, data curation, formal analysis, validation, visualisation, methodology, writing—original draft, writing—review and editing. Yang Chen: Investigation and writing—review and editing. Guofeng Ma: Investigation, validation, resources and writing—review and editing. Sicong Xu: Data curation and formal analysis. Xiangyan Zhang: Data curation and methodology. Lianpeng Chang: Resources and writing—review and editing. Ping Yang, Yujing Xiao, Xuefeng Xia, Shukun Zhang, Huaxiao Tang, Yilin Mou, Lina Zhang: Resources. Haoyan Wang: Writing—review and editing. Jing Bai: Conceptualisation and writing—review and editing. Xin Yi: Resources and supervision. Xiaoming Xing: Conceptualisation, resources, supervision, project administration, writing—review and editing. All authors read and approved the final version of the manuscript. Huiqing Jia and Guofeng Ma accessed and verified the underlying data.

## Data sharing statement

The intermediate data underpinning the study's findings are fully detailed in the supplementary materials. The DNA methylation and transcriptomic profiling data, which have been subject to standardised quality control protocols, are available free of charge from the first author, Jia Huiqin (email: jhq199505@126.com), upon publication of this article. The raw sequencing data generated in this study are currently archived at our collaborating institution, GenePlus-Beijing Institution. Requests for access to raw sequencing data for legitimate academic purposes will be approved following the completion of ethical review and the signing of data authorisation agreements by all involved parties. To request access, applications should be submitted via the institution's platform (https://www.geneplus.cn/contactUs). Alternatively, assistance may be requested from the corresponding author, Xiaoming Xing (email: xiaoming.xing@qdu.edu.cn).

## Declaration of interests

The authors declared no competing interests.
